# SARS-CoV-2 promotes microglial synapse elimination in human brain organoids

**DOI:** 10.1038/s41380-022-01786-2

**Published:** 2022-10-05

**Authors:** Samudyata, Ana O. Oliveira, Susmita Malwade, Nuno Rufino de Sousa, Sravan K. Goparaju, Jessica Gracias, Funda Orhan, Laura Steponaviciute, Martin Schalling, Steven D. Sheridan, Roy H. Perlis, Antonio G. Rothfuchs, Carl M. Sellgren

**Affiliations:** 1 Department of Physiology and Pharmacology, Karolinska Institute, Stockholm, Sweden; 2 Department of Microbiology, Tumor and Cell Biology, Karolinska Institute, Stockholm, Sweden; 3 Department of Molecular Medicine and Surgery, Karolinska Institutet and Center for Molecular Medicine, Karolinska University Hospital, Stockholm, Sweden; 4 Center for Genomic Medicine and Department of Psychiatry, Massachusetts General Hospital, Boston, MA USA; 5 Centre for Psychiatry Research, Department of Clinical Neuroscience, Karolinska Institutet & Stockholm Health Care Services, Stockholm County Council, Karolinska University Hospital, Stockholm, Sweden

**Keywords:** Neuroscience, Cell biology

## Abstract

Neuropsychiatric manifestations are common in both the acute and post-acute phase of SARS-CoV-2 infection, but the mechanisms of these effects are unknown. In a newly established brain organoid model with innately developing microglia, we demonstrate that SARS-CoV-2 infection initiate neuronal cell death and cause a loss of post-synaptic termini. Despite limited neurotropism and a decelerating viral replication, we observe a threefold increase in microglial engulfment of postsynaptic termini after SARS-CoV-2 exposure. We define the microglial responses to SARS-CoV-2 infection by single cell transcriptomic profiling and observe an upregulation of interferon-responsive genes as well as genes promoting migration and synapse engulfment. To a large extent, SARS-CoV-2 exposed microglia adopt a transcriptomic profile overlapping with neurodegenerative disorders that display an early synapse loss as well as an increased incident risk after a SARS-CoV-2 infection. Our results reveal that brain organoids infected with SARS-CoV-2 display disruption in circuit integrity via microglia-mediated synapse elimination and identifies a potential novel mechanism contributing to cognitive impairments in patients recovering from COVID-19.

## Introduction

A significant proportion of patients infected with severe acute respiratory syndrome coronavirus 2 (SARS-CoV-2) display acute neurological and psychiatric symptoms [1], and a subset displays widespread disruptions to micro-structural and functional brain integrity in the recovery stages [2]. Several studies have also reported increased incidence of neurological or psychiatric symptoms after a COVID-19 infection [3–5]. Long-lasting neurobehavioral and cognitive impairments are then common features of other neuro-invasive RNA viruses, with the CNS-damaging processes also continuing after virus elimination [6]. So far, the mechanisms causing such neurocognitive sequelae are poorly understood, but recent studies in rodents suggest that interferon-responses in microglia, the innate immune cells of the brain parenchyma, can lead to excessive microglial synapse elimination and disruption of neuronal circuit integrity [7, 8]. To some extent, this could explain why the developmental period of infection influences the extent of neurocognitive sequelae [9], as microglial functions involving circuit refinement exhibit a clear temporal pattern during neurodevelopment.

Human brain organoids have been successfully employed to study neurotropism and neurotoxic effects of viruses such as the Zika virus [10, 11], and now more recently SARS-CoV-2 [12–16]. However, as microglia are of non-ectodermal origin [17, 18], brain organoids typically lack developing microglia. In this study, we address this limitation by using a newly established protocol for generating undirected brain organoids with innately developing microglia [19], and study cellular responses to SARS-CoV-2 by using single cell RNA sequencing (scRNA-seq) combined with functional characterizations.

## Materials and methods

### Ethics

All individuals signed a written informed consent before participating in the study, as approved by the Institutional Review Board of Partners HealthCare (Boston, MA, USA) and the Regional Ethical Review Boards in Stockholm, Sweden.

### iPSC reprogramming and brain organoid cultures

Two healthy human iPSC lines (males) were used and iPSC mRNA reprogramming was performed as described previously [20]. Undirected brain organoids were prepared from single cell suspension of human iPSCs as previously described [19] but with some modifications (Supplementary Methods).

### Induced microglia-like cells

iMGs were derived from monocytes (donated from one healthy male) using established methods previously described in detail [20, 21].

### Virus isolate, infections, and plaque-forming unit assay

We used original live SARS-CoV-2 (GenBank: MT093571.1). 3 × 10^5^ PFU/ml of SARS-CoV-2 was used to infect organoids for 2 h (the estimated MOI was 1 for periphery and 0.3 for the whole organoid). For scRNA-seq experiments, the viral load was reduced to 1 × 10^5^ PFU/ml (MOI 0.1). PFU assays was performed on 24-well cell culture plates seeded with 2 × 10^5^ Vero E6 cells per well and are described in detail in Supplementary Methods.

### Immunohistochemistry and image quantification

Immunofluorescence images were acquired using Zeiss LSM-800 confocal or a LSM900-Airy*2* (super-resolution) microscope and analyzed using CellProfiler and ImageJ. For more details regarding IHC and quantifications, see Supplementary Methods.

### Quantitative PCR (qPCR)

RNA extraction was performed using DirectZol RNA-Miniprep Kit (Zymo Research Inc.). Quality and concentration of extracted RNA were determined using NanoDrop (Thermofischer Scientific). Total RNA was reverse-transcribed to cDNA using the High-Capacity RNA-to-cDNA Synthesis kit (Thermofischer Scientific). PCR reactions were performed using the StepOnePlus™ Real-time PCR system (Applied Biosystems, Thermofischer Scientific) with PowerTrack™ SYBR Green Master Mix. Relative expression levels in cell lysates were normalized against two housekeeping genes. For more details, see Supplementary Methods.

### Single-cell RNA sequencing

Two single-cell suspensions per condition were loaded onto a single Chromium controller chip v3.1 (10X Genomics), with a target output of 6000–7000 cells per channel. Data processing and analyses are described in detail in Supplementary Methods.

### Statistics

The assumptions of each used test were checked. All reported p-values are two sided and type of statistical test is reported in the figure legends or in the main text.

## Results

### Limited neurotropism but extensive synapse loss in infected brain organoids

**Fig. 1:**
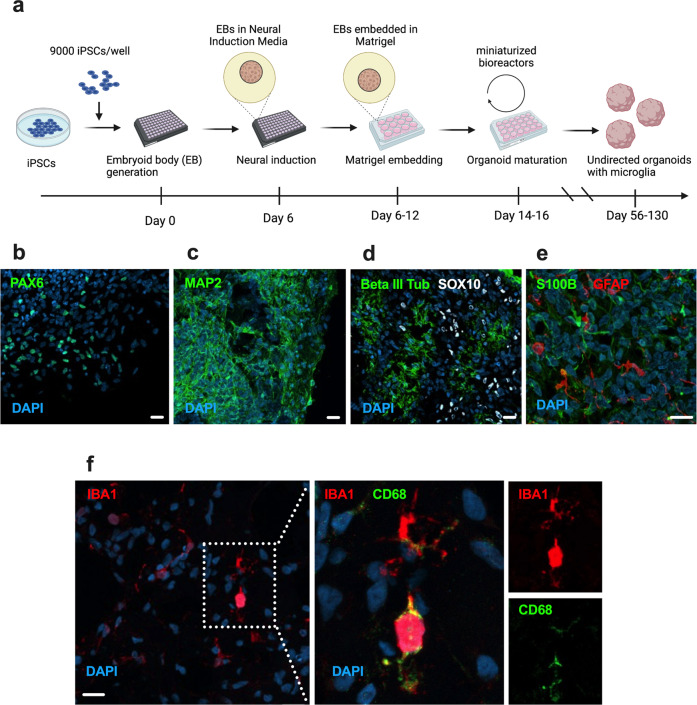
Cellular composition of DIV 56 brain organoids containing innately developing microglia. **a** Schematic of brain organoid generation using an undirected protocol adapted from Ormel et al. [19], **b–e** Representative confocal images (40×) of different cell type markers in organoid cryosections showing the presence of NPCs (PAX6), immature and mature neurons (Beta III Tubulin and MAP2), neural crest cells (SOX10), astrocytic lineage cells (GFAP, S100B) and **f** microglia (IBA1, CD68). Nuclei are counterstained with 4′,6-diamidino-2-phenylindole (DAPI) (blue). Scale bar for representative images in the panel: 20 μM.

With a few modifications, the protocol reported by Ormel et al. [19] was adapted to miniaturized bioreactors (Fig. 1a). After 56 days in vitro (DIV), these undirected organoids were found to contain neural progenitor cells, neurons, neural crest cells, cells of astrocytic lineage, and microglia (Fig. 1b–e). We observed no convincing staining for AQP4 (indicating a lack of mature astrocytes) or TMEM119 (suggesting a more fetal microglial profile). To confirm differentiation trajectories towards maturation in the model, we performed additional staining at 130 DIV and observed a general decrease in expression of progenitor markers (Supplementary Fig. 1a, b). In addition, we could detect AQP4^+^ astrocytes (Supplementary Fig. 1c, d) and a small subset of IBA1^+^ microglia that also expressed TMEM119 (Supplementary Fig. 1e). Nonetheless, mRNA expression of *TMEM119* was low compared to other microglial markers (Supplementary Fig. 1f), then suggesting that microglia at 130 DIV still displayed a more fetal profile [22].

**Fig. 2:**
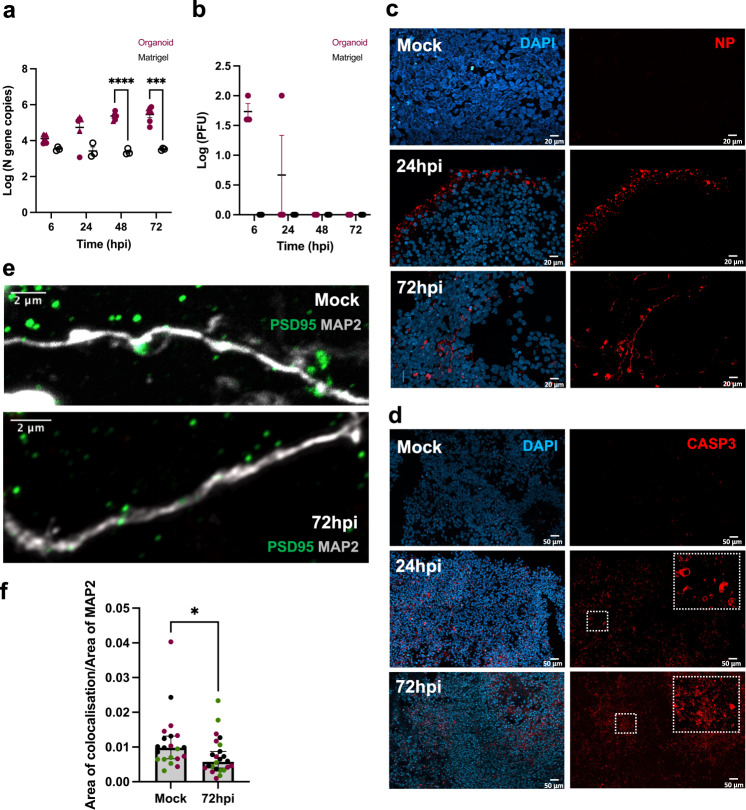
SARS-CoV-2 infection of immunocompetent brain organoids. Brain organoids and Matrigel droplets were harvested at multiple time points following exposure to live SARS-CoV-2 (MOI 0.3). **a** qPCR analyses on supernatants from infected Matrigel droplets (*n* = 3, black circle) and brain organoids corresponding to 56 DIV (*n* = 3, magenta triangle) and 130 DIV (*n* = 3, magenta circle), showing log fold change in viral nucleocapsid (*N)* gene copies at different hours post infection (hpi). Error bars indicate standard errors of the mean (SEM). Data were analyzed with a 2-way repeated ANOVA model. Post-hoc *p* values for comparing organoids to Matrigel at 48 hpi and 72 hpi: *P* < 0.0001 and *P* = 0.004, respectively. Center values represent means and error bars represent S.E.M. **b** Log transformed PFUs of supernatants from infected brain organoids (*n* = 3, magenta circle) and Matrigel droplets (*n* = 3, black circle). Error bars indicate SEM. Tiled confocal images (40×) showing immunostaining of **c** nucleocapsid protein (NP) and **d** cleaved caspase 3 (CASP3) in 56 DIV infected brain organoids and mock-treated organoids. Nuclei counterstained with DAPI. Scale bars for representative images in **c**: 20 μM and **d**: 50 μM. **e** Super resolution confocal image (Airyscan, 63×) showing immunostaining for postsynaptic density (PSD-95) on neurons (MAP2) from mock-treated and 72hpi conditions. Scale bar for representative images: 2 μM. **f** Quantification of co-localization index (Area of colocalization between PSD-95 and MAP2 channels normalized by area of MAP2 channel; 63×) shown in mock-treated and 72hpi organoids. Each datapoint represents the mean of all cells per field of view (FOV) and is stratified by organoid (*n* = 3). Significant decrease in post-synaptic density at 72hpi (*P* = 0.019) generated by a Mann-Whitney *U*-test. Center values represent medians and error bars represent 95% CIs. All reported *p* values in the figure are two-sided. *P < 0.05, **P < 0.01, ***P < 0.001.

We exposed organoids (56 DIV) to active SARS-CoV-2 virus for 2 h at an estimated MOI of 0.3, after which they were washed and transferred into fresh media to monitor the course of infection. Matrigel droplets were used as control to account for unspecific virus capture and release. Supernatants were harvested at 6, 24, 48, 72 h post-infection (hpi). Viral transcription and release measured in supernatants via RT-qPCR showed a time dependent increase of SARS-CoV-2 transcripts in organoid supernatants compared to those from Matrigel droplets but at a slower pace as compared to Vero-E6 cells (Fig. 2a; Supplementary Fig. 2a). To validate that SARS-CoV-2 could assemble and release complete viral particles in the organoids, we performed plaque formation assays and observed release of infectious viral particles as early as 6 hpi, although the number of plaque forming units (PFUs) then quickly decreased (Fig. 2b). This suggests that the limited number of infected cells either have an inefficient assembly and shedding of viral particles, or that cell death decreases the availability of viable cells to sustain the production of fully assembled viral particles.

Based on previous literature [12–16], we decided to fix infected and mock-treated organoids (56 DIV) for immunohistochemical (IHC) analyses at two-time points (24 and 72hpi). At 24hpi, a strong signal for the viral nucleocapsid protein (NP) was observed mainly in the periphery of the infected organoids, whereas at 72hpi, we observed NP staining in cellular cytoplasm or processes of infected cells (Fig. 2c; Supplementary Fig. 2b). Cleaved caspase3 (CASP3) staining indicated a pronounced increase in early cell death-related events in infected organoids as compared to mock-treated organoids (Fig. 2d). Casp3^+^ cells were not limited to superficial layers and exceeded the number of NP^+^ cells at both time points (Supplementary Fig. 2b). Given previous reports [7, 8] that other RNA viruses can induce long-lasting interferon-responses in microglia that lead to excessive microglial synapse elimination and disruption of neuronal circuit integrity post infection, we also decided to evaluate synapse density at 72hpi. Infected organoids then displayed a significant decrease in postsynaptic density (PSD-95) as compared to the mock-treated organoids (Fig. 2e, f).

**Fig. 3:**
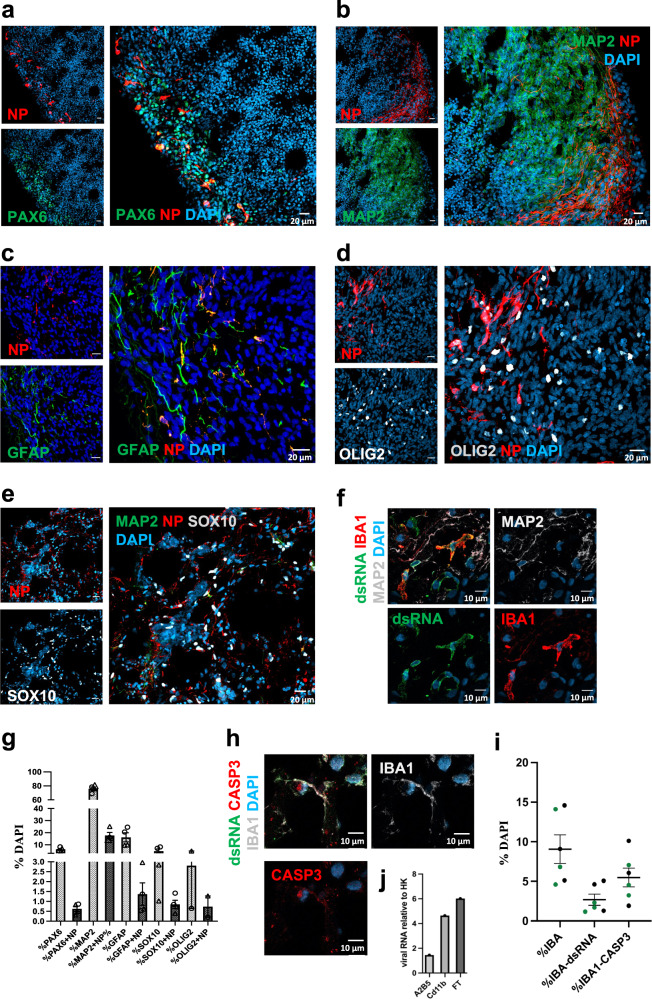
Cell type specific SARS-CoV-2 infection in brain organoids with innately developing microglia. Confocal images (40×) at 72hpi (MOI 0.3) showing colocalization of viral nucleoprotein (NP) with **a** neuronal precursors (PAX6), **b** neurons (MAP2), and a heterogenous population of progenitors marked by **c** GFAP **d** OLIG2 and **e** schwann cell precursors (SOX10). Scale bars for representative images **a**–**e**: 20 μM. Confocal images (40×) showing colocalization of organoid grown microglia (IBA1) with **f** dsRNA (clone 9D5, for viral presence). Nuclei stained with DAPI. Scale bar for **f**: 10 μM. **g** Distribution of NP^+^ cells amongst different cell-type markers, expressed as percentage of DAPI from two infected organoids at DIV 56 (corresponding data points indicated by circles and triangles). Each data point is an average of four FOVs representing different areas on the same section. Error bars indicate SEM. **h** Confocal images (40×) showing colocalization of organoid-grown microglia (IBA1) with CASP3 (obtained from one donor). Nuclei stained with DAPI. Scale bar for **h**: 10 μM. **i** Quantification of dsRNA and CASP3 presence in IBA1^+^ cells expressed as percentage of DAPI from two infected organoids at DIV 56 **j** qPCR on cell lysate fractions obtained after magnetic cell sorting (MACS) with CD11b beads, (also shown A2B5 radial glia enriched fraction) and flowthrough (FT), on infected organoids (*n* = 2, pooled) showing presence of viral N gene copies normalized to two housekeeping host genes. Center values represent means and error bars represent S.E.M.

Next, we investigated infectivity and cell death in the context of cellular identity. IHC staining on infected organoids at 72hpi showed viral NP or dsRNA overlapping with PAX6^+^, MAP2^+^, GFAP^+^, SOX10^+^, OLIG2^+^, and Iba1^+^ cells (Fig. 3a–f). Both NP^+^ and CASP3^+^ staining was foremost observed in neurons, but also included other cell types (Fig. 3g–i and Supplementary Fig. 2c, d). Additionally, we enriched microglial cells from two infected organoids. Consistent with our previous microglial characterization (IHC), qRT-PCR on cell lysates suggested the capture of more immature microglia (Supplementary Fig. 2e) and included viral *N* gene copies (Fig. 3j). To exclude those microglia that only stained positive for viral RNA (dsRNA, *N* gene), and/or CASP3, due to engulfment of infected cells within the organoid, we derived induced human microglia-like cells (iMGs) in 2D-culture [20] from one donor and exposed these cells to live SARS-CoV-2 virus (using a lower MOI of 0.01 given monoculture). The proportion of IBA1^+^ cells positive for dsRNA and CASP3 then increased with time (Supplementary Fig. 2f, g).

### Increased microglial engulfment of postsynaptic termini in infected organoids

**Fig. 4:**
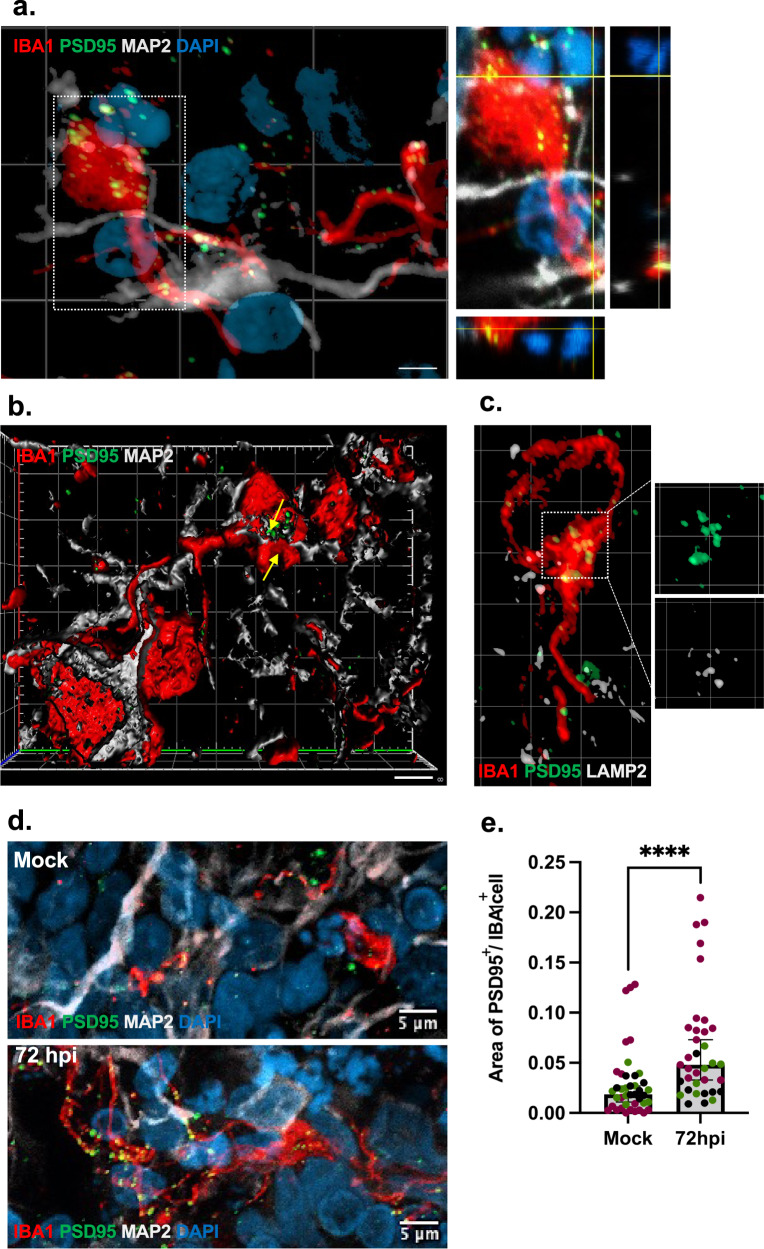
Microglial engulfment of synaptic material in response to SARS-CoV-2 exposure. Super resolution confocal (Airyscan, 63×) images showing **a** microglial processes (IBA1^+^) in proximity to neurons (MAP2^+^), engulfing post-synaptic material (PSD95^+^) with orthogonal projections showing PSD95 overlapping with IBA1 **b** 3D reconstruction and surface rendering demonstrating volumes of PSD-95^+^ puncta inside IBA1^+^ cells (indicated by arrows). **c** postsynaptic material (PSD95^+^) colocalizing with lysosome-associated membrane protein, LAMP2, within phagocyting microglia (IBA1^+^). Scale bars for **b**, **c**: 2 μM. **d** Confocal images (Airyscan, 63×) showing microglial engulfment of post-synaptic material (PSD-95^+^) in mock-treated and infected organoids (MOI 0.3) at 72hpi. Nuclei are stained with DAPI. Scale bars for representative images: 5 μM. **e** Quantification of phagocytic index (area of PSD-95 puncta normalized by area of IBA1^+^ cell) shown in mock-treated and 72hpi organoids. Two lines were used and one of the lines include both 56 and 130 DIV organoids. Each datapoint represents the mean of all cells per field of view (FOV) and datapoints derived from separate organoids are indicated. We observed a significant increase in microglial engulfment of post-synaptic material (PSD-95^+^) in infected organoids (Mann-Whitney *U*-test; *P* < 0.0001). Phagocytic index also predicted the infected condition in a binary logistic regression adjusting for organoid age and line *(P* < 0.001) while no significant effect was observed for organoid age or line. Reported *p* value are two-sided. ****P < 0.0001. Center values represent medians and error bars represent 95% CIs.

To determine if SARS-CoV-2 has the capability to induce microglial phagocytosis of synaptic structures and contribute to the observed decrease in postsynaptic density observed in infected organoids, we first investigated to what extent microglia contributed to synapse remodeling in mock-treated organoids. In line with our previous findings in 2D models [20], this revealed a baseline uptake of postsynaptic structures (indicated by PSD-95) in microglia residing in brain organoids (Fig. 4a–c). In infected organoids (MOI 0.3, 72hpi), the total numbers of Iba1^+^ cells were slightly increased but did not reach significance (Supplementary Fig. 3a) while we observed a clear increase in CD68^+^ cells (Supplementary Fig. 3b) and a threefold increase in uptake of synaptic structures in microglia (Fig. 4d, e). After viral exposure, microglia cells also displayed a subtle but significant structural change that indicated retraction of fine processes and a transformation to a less ramified morphology (Supplementary Fig. 3c–g).

### Single-cell characterization of brain organoids containing resident immune cells

**Fig. 5:**
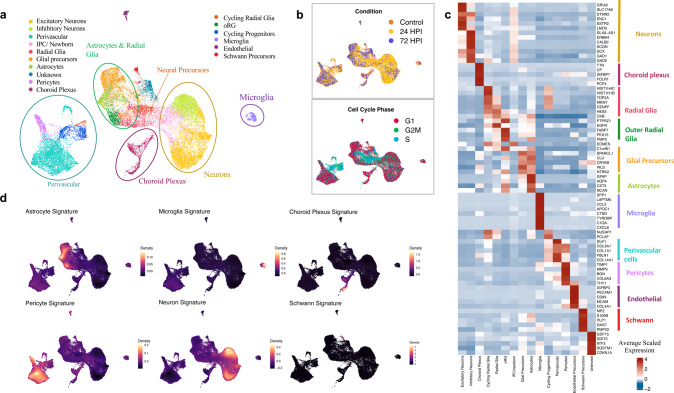
Single-cell transcriptome profiling of brain organoids with innately developing microglia. DIV 130 brain organoids (*n* = 3, MOI 0.1) per experimental condition (Mock-treated, 24 and 72hpi) were dissociated into whole-cell suspensions and single-cell RNA sequencing libraries were generated using the droplet-based 10× chromium platform. **a** UMAP plot of integrated dataset containing 25336 single cells from all three conditions depicting the presence of key neurodevelopmental cell types. Individual dots representing single cells are colored by the identified cell type. **b** UMAP plot of overall embedding of cells colored by the experimental condition (top) and cell cycle phase (bottom) **c** Heatmap showing expression of top-differentially expressed markers (rows) across all clusters (columns) with a two-sided Benjamini–Hochberg corrected *p* value < 0.05. Colored column bars (right) highlight the identified cellular groups. A list of all differentially expressed genes conserved across conditions for each cluster is provided in Supplementary Table 1. **d** Cell type classification obtained by scoring cells by their expression of known individual cell type signatures (Supplementary table 1) plotted as estimated joint density on a UMAP.

Droplet-based encapsulation of single cells allowed us to profile and compare transcriptomes of individual cells isolated from SARS-CoV-2 infected brain organoids as compared to mock-treated organoids. Given the extent of cell death in the organoids exposed to an estimated MOI of 0.3, we then decreased the MOI to 0.1, and to also capture more mature cells we used organoids cultured up to 130 DIV and enriched for CD11b^+^ cells (MACS). In this way we were able to obtain good-quality transcriptomic data from fresh cells isolated in three experimental conditions: mock-treated organoids (7257 cells), 24hpi organoids (15254 cells), and 72hpi organoids (2825 cells), respectively (Supplementary Fig. 4a–d). Then, we integrated pre-processed data from each condition and performed unsupervised graph-based clustering to obtain cellular clusters with similar transcriptomic identities across conditions (Fig. 5a, b, Supplementary Fig. 4e–i). Supervised inspection of top differentially expressed genes (DEGs) per cluster combined with cell type-specific gene signatures and cluster correlations to other developing human brain [23–29], as well as organoid [30–32], datasets confirmed 16 clusters (Fig. 5c, d, Supplementary Table 1, Supplementary Fig. 5 and Supplementary Fig. 6a–e). To address if organoid-grown microglia at 130 DIV more resembled adult or fetal primary microglia, we performed integration of single-cell RNA sequencing data from our microglia cluster with two primary fetal microglia datasets [23, 27], as well as a primary adult dataset [28], and concluded that the overall transcriptomic profile was closest to fetal microglia (Supplementary Fig. 6f).

We then assessed the expression of previously identified entry factors for SARS-CoV-2 and observed basal RNA expression for most factors although the relative expression was rather low (Supplementary Fig. 6g–i, and Supplementary Results for further details). Next, we identified infected cells by aligning cellular viral transcripts to the whole SARS-CoV-2 genome in the infected conditions. The percentage of infected cells was low (0.1–0.2% of sequenced cells), which was expected given the lower MOI and to some extent the removal of non-viable infected cells, although we were able to qualitatively identify infected neurons, radial glia, astrocytes, and choroid plexus cell types (Supplementary Table 2). Amongst infected cells, we observed a preferential infection of neurons (*p* = 0.014) as well as radial glia (*p* = 0.002). For further downstream analyses, we then did not make any distinction between infected and non-infected cells, instead focusing on overall cellular responses in the infected conditions.

### Interferon-responsive microglia with upregulation of genes promoting synapse elimination

**Fig. 6:**
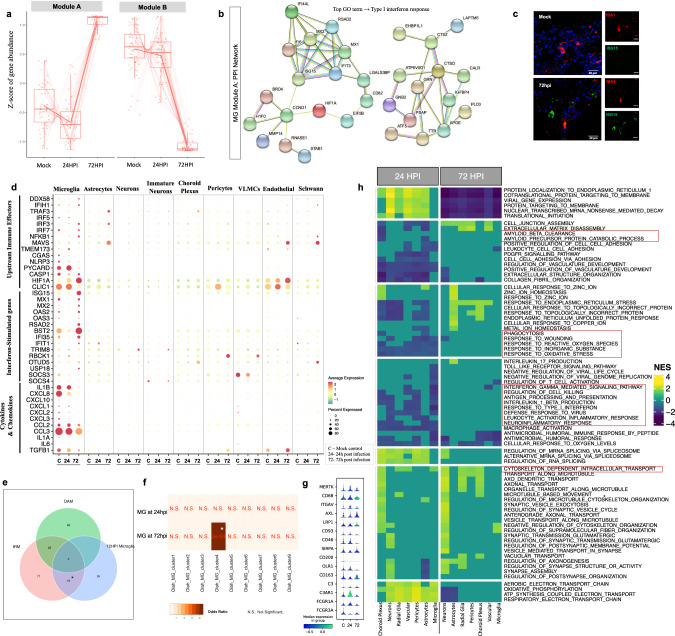
Expression of SARS-CoV-2 entry factors in brain organoids and microglial responses to SARS-CoV-2. **a** Modules of genes with similar expression behavior across conditions in microglia identified by pseudo-bulk differential gene expression analysis (using LRT implemented in DESeq2). **b** Protein-protein interaction network of genes belonging to module A using STRING database with the top Gene Ontology term identified as ‘Type I interferon response’ (two-sided Benjamini-Hochberg corrected *p* value < 0.05). **c** Confocal images (40×) validating ISG15 expression in control and 72hpi organoid at DIV 130. Quantifications provided in supplementary Fig. 6c. Scale bars for representative images: 20uM. **d** Dot plot showing expression levels of neuro-immune related genes such as cytokines & chemokines, interferon-stimulated genes, and their upstream effector molecules across infected conditions for each cell type [C- mock-treated; 24–24 hpi; 72–72 hpi]. Color scale represents average log-scaled expression values across all cells, in each cluster. Size of the dot represents the percentage of cells in each cluster, expressing the gene. **e** Venn diagram of genes overlapping between gene signatures of Disease-associated microglia (DAM), Injury-associated microglia (IRM), and SARS-CoV-2 infected microglia at 72hpi (* two-sided Benjamini-Hochberg corrected *p* value < 0.005). No significant overlap was observed at 24hpi. **f** Heatmap of comparisons between SARS-CoV-2-associated microglia at 24hpi and 72hpi along with 9 defined human microglial clusters [38], colored by odds ratio (with two-sided Bonferroni corrected p-values shown in red) from hypergeometric gene overlap testing (Fischer’s exact test). Transcriptomic gene signatures were defined as differentially upregulated genes with log2FC > 0.25 and FDR < 5%. **g** Violin plot showing normalized expression of microglial genes associated with active phagocytic states across conditions. **h** Heatmap showing gene set enrichment analyses (*MSigDB* GO:BP gene sets) of differentially expressed genes (DEGs) from 24hpi versus mock-treated (left) and 72hpi versus mock-treated (right) for each cell type. Pathways commonly dysregulated across most cell types were selected. All significantly altered pathways for individual cell types are listed in Supplementary Table 4 (FDR < 5%, Benjamini–Hochberg correction, NES- Normalized Enrichment Score).

To define microglial responses to SARS-CoV-2 exposure, we first performed pseudo-bulk differential gene expression testing across the three experimental conditions. An unbiased hierarchical clustering approach generated two unique groups of DEGs (labeled modules A and B). Module A comprised of genes whose expression on average decreased at 24hpi and then at 72hpi displayed a more pronounced increase, while module B genes on average displayed reduced expression at 24hpi and a more dramatic decrease at 72hpi (Fig. 6a). While module B was largely comprised of genes encoding for cytosolic ribosomal proteins (Supplementary Fig. 7a, b), we detected several interferon-stimulated genes (ISGs) in module A (Fig. 6b, Supplementary Table 3). Accordingly, IHC validation of ISG15 protein showed a general increase in interferon response in organoids at 72hpi as well as a specific upregulation in IBA1^+^ cells, compared to control mock organoids (Fig. 6c, Supplementary Fig. 7c). Nonetheless, we detected no clear upregulation of pro-inflammatory cytokines in microglia or microglial subclusters (Supplementary Fig. 7d, e), at either 24hpi or at 72hpi, although several pathogen sensors and upstream effectors of interferon signaling were upregulated including nuclear factor kappa B (*NFKB1*) (Fig. 6d). At 24hpi, we detected upregulation of pathways (Supplementary Table 4) that can serve as activating stimuli for primed inflammasome components, and are required for the production and maturation of pro-inflammatory cytokines (Fig. 6d) [33]. Taken together, this suggests that SARS-CoV-2 activates interferon signaling in microglia although the expression of pro-inflammatory cytokines are at normal levels already at 24hpi.

In the infected conditions, unbiased analyses revealed a significant upregulation of pathways related to neurodegenerative diseases, including Alzheimer’s disease (AD) and Parkinson disease (PD) (Supplementary Fig. 7f). Notably, both these disorders are characterized by a microglia-mediated early synapse loss [34], and epidemiological register studies have indicated an increased risk of dementia and parkinsonism after COVID-19 infection [4]. This led us to examine the microglial transcriptional state more closely after exposure to SARS-CoV-2 in relation to previously described disease-associated microglial activation states. First, we compared SARS-CoV-2-exposed microglia to such activation states observed in experimental murine models. Injury-responsive microglia (IRM) have been described in mice and are to a large extent defined by upregulation of ISGs [35, 36], while the disease-associated microglia (DAM) described in mouse models of AD and ALS are thought to restrict neurodegeneration and disease development [37]. SARS-CoV-2 exposed microglia at 72hpi (although not at 24hpi; data not shown) then displayed a significant enrichment for genes associated with the IRM activation state (*p* = 6.9 × 10^−24^, Fig. 6e, Supplementary Fig. 7g), and less so for the unique DAM signature genes (*p* = 0.11), and the significant IRM-intersecting signature enriched for genes found in the “Coronavirus disease” pathway (Supplementary Fig. 7g). However, microglia displayed upregulation of genes implicated in AD with a non-significant decrease of core microglial homeostasis genes (e.g., *P2RY12* and *CX3CR1*; Supplementary Fig. 7h). In fact, several of these genes were observed to be commonly dysregulated across all three signatures (IRM, DAM and SARS-CoV-2 exposed microglia), suggesting unique as well as shared activation mechanisms across these microglial states. Further, we compared the SARS-CoV-2 microglial transcriptomic signature to previously identified human microglial populations identified in autopsy and surgical brain tissues obtained in the Memory and Aging Project (MAP) [38], and found an enrichment with genes in so-called interferon responsive microglia (cluster 4 in this dataset) (Fig. 6f), defined by *ISG15* and with increased expression of multiple sclerosis (MS) and AD susceptibility genes. To exclude effects of tissue processing, we also compared microglia from the organoids with a cluster defined by general cellular distress due to tissue processing (cluster 3) but observed no enrichment (Fig. 6f).

Actin-cytoskeletal remodeling pathways, essential for promoting migration and phagocytosis [39], were also upregulated in microglia exposed to SARS-CoV-2 (Fig. 6g, h, Supplementary Fig. 7f). Consistent with this upregulation in microglia, and the extensive initiation of neuronal apoptosis and decreased postsynaptic density in infected organoids, neurons at 72hpi downregulated the expression of ‘don’t-eat-me’ signals such as *CD46* and *CD200* (Supplementary Fig. 7i) [40]. Consequently, we observed increased microglial expression of genes associated with microglia-mediated phagocytosis [41–44], such as *CD68* (in line with the observed increase of CD68^+^ cells)*, TREM2*, *ITGB5*, *CD47, MSR1, CALR*, as well as genes previously identified to be directly involved in synapse elimination in the aftermath of viral encephalitis, such as *C3*, *C3AR1* and *FCGR3A* [6] (Fig. 6g).

### Astrocytic subclusters with enrichment for genes implicated in neurodegenerative diseases

**Fig. 7:**
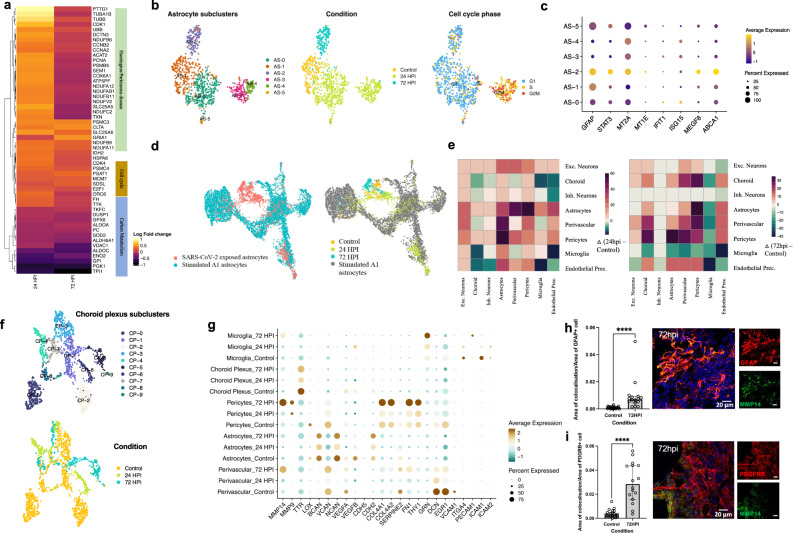
Temporal heterogeneity in astrocytes and choroid plexus responses to SARS-CoV-2. **a** Differential expression analysis of SARS-CoV-2 exposed astrocytes versus control astrocytes, highlighted DEGs in a heatmap with top implicated pathways using overrepresentation test (FDR < 0.05, Benjamini-Hochberg correction; Scale bar- Log2fold change values). **b** Cells belonging to the astrocyte cluster, as shown in Fig. 5a, were extracted and subjected to further unsupervised clustering. UMAP plot of astrocytes shows six unique subclusters (AS-0, AS-2, AS-2, AS-3, AS-4 & AS-5; left) with cells colored by condition (middle) and cell cycle phase (right) (clustering was performed upon regressing cell cycle effects). Each dot represents a single cell colored by the respective groups shown on the right side of each plot. **c** Dot plot of scaled average expression of genes involved in astrocytic engulfment across astrocyte subclusters (adjusted *P* < 0.05) **d** UMAP plot of single-cell transcriptomic data of human iPSC-derived A1-astrocytes [47], integrated with astrocytes from our study using canonical-correlation based-approach (CCA) (implemented in Seurat). Cells are colored by their respective dataset and experimental condition. **e** Mapping intercellular interactions across cell types upon infection. Heatmap showing changes in the number of interactions between cell types at 24hpi (left) and 72hpi (right) resulting from SARS-CoV-2 infection. Ligand-receptor interactions were inferred in the single-cell transcriptomic data using *CellphoneDB*. Green depicts a negative difference indicating loss of interactions whereas purple depicts a positive change indicating gain of interactions between the cell type pair when compared to the control. **f** Cells belonging to the choroid plexus cluster, as shown in Fig. 5a, were extracted, and subjected to further unsupervised clustering. UMAP plot of choroid plexus shows 9 unique subclusters (top) with cells colored by condition (bottom). Each dot represents a single cell colored by the respective groups shown on the right side of each plot. **g** Dot plot showing Average**-**scaled expression of genes related to blood-brain-barrier integrity and function for different cell type clusters across control and infected conditions. **h** Confocal images (40X) and corresponding quantification validating increased MMP14 expression in GFAP^+^ cells and **i** PDGFRB^+^ cells (*P* < 0.0001, generated by a Mann-Whitney *U* test. Center values represent medians and error bars represent interquartile ranges. All reported *p* values in the figure are two-sided. *P < 0.05, **P < 0.01, ***P < 0.001, *P* < *0.0001*). Scale bars for **h** and **i**: 20 μM.

In response to SARS-CoV-2, astrocytes displayed DEGs enriched for mechanisms involved in cell cycle, carbon metabolism and disorders such as Huntington’s disease (HD) and PD (Fig. 7a). Already at 24hpi, three subclusters of astrocytes could be identified: AS-0, AS-3 and AS-4 (Fig. 7b, Supplementary Fig. 8a, b). AS-0 cells expressed relatively higher levels of anti-viral ISGs, while AS-3 and AS-4 cells had a proliferative profile and exhibited lower expression levels of *GFAP* (Fig. 7b, c, Supplementary Fig. 8c). Subcluster AS-2, comprising of cells sampled at 72hpi, showed elevated levels of *GFAP* and *STAT3*, indicative of reactive astrogliosis [45], along with phagocytosis related genes such as *MEGF8* and *ABCA1* (Fig. 7c, Supplementary Fig. 8d). We also observed upregulation of metal ion (zinc, copper, iron) homeostasis pathways involving metallothioneins in AS-2 astrocytes at 72hpi (Fig. 7c). Notably, proliferative reactive astrocytes (AS-3 and AS-4) appeared mainly at 24hpi but were largely replaced with non-proliferative reactive astrocytes at 72hpi, then in line with the microglial signature suggesting an early switch to a more chronic reactivity state [46].

In neurodegenerative diseases, astrocytes have been shown to express markers of a putative reactive neurotoxic state commonly referred to as A1 [46]. We then integrated scRNA-seq data from A1 astrocytes (stimulated with IL-1A, TNF-α, and C1q) [47], and SARS-CoV-2 exposed astrocytes via canonical correlation analysis (CCA). Although the majority of SARS-CoV-2 exposed astrocytes (AS-0, AS-1, AS-2) clustered separately from A1 astrocytes, the proliferative clusters (AS-3 and AS-4), as well as a subset of AS-0 astrocytes, showed clustering similarities with a portion of A1 astrocytes (Fig. 7d, Supplementary Fig. 8e, f). This is in line with a decreased phagocytic capacity of A1 astrocytes [48], and an upregulation of phagocytosis-related genes in the AS-2 cluster. Furthermore, higher expression levels of metallothioneins, as in AS-2 cluster (72hpi), have been observed in astrocytes from HD patients [49].

### Secretome alterations indicate a compromised capacity to keep BBB integrity

Potential entry routes to the CNS for SARS-CoV-2 include the blood–brain barrier (BBB) or the blood–cerebrospinal–fluid-barrier, involve cell types such as endothelial cells, pericytes, astrocytes and choroid plexus-epithelium. Upon encountering pathogens, these barrier cells act in concert to activate and regulate signal transduction pathways that aid invasion of peripheral immune cells and restoring of CNS homeostasis [50]. Correspondingly, at 24hpi we found increased ligand-receptor communication between astrocytes, neurons, microglia, perivascular, and endothelial cells, whereas at 72hpi, both microglia and neurons significantly reduced their communication with most other cell types, while choroid plexus cell types gained interactions (Fig. 7e). Sub-clustering of choroid plexus related cells (Fig. 7f, Supplementary Fig. 9a, b) showed two control enriched clusters (CP-0 and CP-2), consisting of genes involved in maintaining solute homeostasis, barrier, or extracellular matrix (ECM) integrity, and amyloid clearan amongst others (Supplementary Fig. 9c). At 24hpi, we observed an enrichment of metallothioneins, similar to what we observed in astrocytes at 72hpi, whereas genes involved in viral defense response, regulation of reactive oxygen species, and ECM organization, were enriched at 72hpi (CP-5) (Supplementary Fig. 9c). In addition to choroid plexus, a differential expression analysis showed significant increase in expression of matrix metalloproteases and ECM-regulatory enzymes at 72hpi in microglia, astrocytes and perivascular cells, whereas endothelial cells reduced expression levels of solute carrier *SLC2A1* (Fig. 7g), all in agreement with signaling that promotes compromised CNS barriers. Accordingly, by IHC we validated increased MMP14 protein expression by PDGFRB^+^ (pericytes) as well as GFAP^+^ cells within 72hpi organoids (Fig. 7h, i). Further, astrocytes, pericytes and choroid plexus-related cells were observed to downregulate VEGF signaling, a potent inducer of BBB permeability, at 24hpi but then at 72hpi instead display an upregulation, coinciding with increased signaling for leukocyte chemotaxis and activation in astrocytes, as well as antigen processing and presentation in choroid plexus (Fig. 7g).

### Metabolic dysregulation across cell types

Protein accumulation in the endoplasmic reticulum (ER) following viral infection or excessive production of secretory proteins can induce ER stress and initiate countermeasures in the form of reduced translation and export to ER along with unfolded protein responses (UPRs) [51]. In exposed organoids, we detected an upregulation of pathways related to UPRs, ER stress, proteasomal degradation, and autophagy, among cell types such as microglia, astrocytes, choroid plexus, and perivascular cells. Further, a partial translational inhibition could be observed in microglia (module B), as well as most of the other cell types. We also observed downregulation of the ROS metabolism across cell types, which can lead to dysregulation of the antioxidant cellular systems and result in oxidative stress [52]. See also Supplementary Results.

## Discussion

Long-lasting neurobehavioral and cognitive impairments have been increasingly recognized in the COVID-19 pandemic [3–5]. After recovery, patients display an increased risk of receiving a neurological or psychiatric diagnosis [6], while imaging studies have revealed widespread micro-structural disruptions in a subset of the recovered patients [2, 3]. To a large extent, the described sequalae overlap with post-infection syndromes described in patients that have recovered from infection with more uncommon neuro-invasive RNA viruses [6]. In murine models for encephalitis caused by such viruses, CNS damage continues after virus elimination [6], and can be largely attributed to interferon-responsive microglia that excessively eliminate synaptic termini as well as induce neuronal apoptosis [7, 8].

Here we infect brain organoids containing developing microglia with live SARS-CoV-2 virus and observe an increase in early cell-death related events. Further, we observe a striking reduction in post-synaptic density and show that microglia in the infected organoids increase the engulfment of postsynaptic termini with a concomitant upregulation of interferon-responsive genes as well as genes promoting phagocytosis and synapse elimination, while neurons down-regulate “don’t-eat-me” signals. While several postmortem studies have observed neuronal cell death [53], none have, to the best of our knowledge, evaluated synapse density and microglial synapse engulfment. However, a recent longitudinal and observational brain imaging study, reported a reduction of grey matter thickness also in non-hospitalized patients post COVID-19 [54], then suggesting that Covid-19 infection causes a reduction in synapse density.

Single-cell transcriptomics revealed that the glial gene signatures in infected condition early adopt a transcriptomic profile overlapping with those observed in neurodegenerative conditions. In line with this, a recent study also observed ApoE-isoform-dependent decreases in neurite length and synaptic loss in SARS-CoV-2 infected neuron-astrocyte co-cultures [55], while gene expression analyses of postmortem material also indicate a similar immune activation overlapping with profiles observed in neurodegenerative disorders [56]. Accordingly, these disorders are characterized by an early synapse loss [34], and the incidence of symptoms overlapping with these disorders is increased after COVID-19 [4]. Similar microglia-mediated synapse elimination has also been observed in schizophrenia models [20], another disorder that displays an increased incident risk after COVID-19 infection [4].

We also observed secretome alterations in astrocytes, pericytes, and choroid plexus-related cells indicating a compromised capacity to keep BBB integrity, as well as an upregulation of pathways related to signaling with peripheral immune cells. Murine models of brain infection with other RNA viruses have revealed a role of infiltrating CD8^+^ T-cells to activate interferon signaling in microglia [8], then suggesting that a similar mechanism could also exacerbate microglial synapse elimination secondary to SARS-CoV-2 exposure.

Existing protocols for generating brain organoids have several constraints that also limit our interpretations. Brain organoids most closely resemble the developing fetal brain rather than the mature adult brain. Thus, we cannot exclude important differences exist in the cellular responses and tropism between immature and more mature brain cells, as our model primarily recapitulates the responses to SARS-CoV-2 of the developing brain. By only including male lines, as well as original SARS-CoV-2 virus, we cannot exclude that using female lines as well as using other SARS-CoV-2 variants could have influenced our results. That said, to the best of our knowledge, there is currently no data suggesting that CNS-related symptoms differ between sexes or SARS-CoV-2 variants. Using a larger number of lines could also have revealed subject-specific differences regarding infectivity and microglial synapse engulfment. Finally, as our cellular characterizations were performed at later time points, we may have missed functional changes directly related to active viral release.

In summary, we here provide an experimental approach for evaluating viral effects on the brain that includes tightly orchestrated responses of microglia and astrocytes in the context of neuronal circuits. Challenging this model with modest live SARS-CoV-2 virus titers, we observe neuronal cell death and microglia-mediated synapse loss. A key next step will be to determine the clinical importance of such molecular processes, and specifically whether they may contribute to synapse loss and the neurocognitive and neuropsychiatric symptoms observed in a subset of COVID-19 patients across different developmental stages. If so, these microglia-containing organoid models may provide an opportunity to evaluate microglia-targeted therapeutics aimed at minimizing or preventing COVID-19 sequelae.

## Supplementary information

Supplementary

Supplementary Figure 1

Supplementary Figure 2

Supplementary Figure 3

Supplementary Figure 4

Supplementary Figure 5

Supplementary Figure 6

Supplementary Figure 7

Supplementary Figure 8

Supplementary Figure 9

Supplementary Table 1

Supplementary Table 2

Supplementary Table 3

Supplementary Table 4

## Data Availability

Processing of data and downstream analysis was performed in R (version 4.0.3). Figures used in this manuscript were generated in Python (version 3.6.12). Raw single-cell RNA sequencing data is deposited into GEO database (GSE181422). Source code to reproduce the findings are available in our repository on Github (https://github.com/SellgrenLab/organoid-Covid19). All other data are available from the corresponding authors upon request.
