# Supplementary material for: SARS-CoV-2 promotes microglial synapse elimination in human brain organoids
Source: Mol Psychiatry. 2022 Oct 5;27(10):3939–50. doi: 10.1038/s41380-022-01786-2 (PMC9533278; doi:10.1038/s41380-022-01786-2)
Supplement: Supplementary file 1 — Supplementary [file 41380_2022_1786_MOESM1_ESM.docx]

**SARS-CoV-2 promotes microglial synapse elimination
in human brain organoids**

Samudyata^1*^, Ana O. Oliveira^1*^, Susmita Malwade^1*^, Nuno Rufino de Sousa^2^, Sravan K. Goparaju^1^, Jessica Gracias^1^, Funda Orhan^1^, Laura Steponaviciute^2^, Martin Schalling^3^, Steven D. Sheridan^4^, Roy H. Perlis^4^, Antonio G. Rothfuchs^2^, Carl M. Sellgren^1,5#^

^1^Department of Physiology and Pharmacology, Karolinska Institute, Stockholm, Sweden.

^2^Department of Microbiology, Tumor and Cell Biology, Karolinska Institute, Stockholm, Sweden.

^3^Department of Molecular Medicine and Surgery, Karolinska Institutet and Center for Molecular Medicine, Karolinska University Hospital, Stockholm, Sweden.

^4^Center for Genomic Medicine and Department of Psychiatry, Massachusetts General Hospital, Boston, MA, USA.

^5^Centre for Psychiatry Research, Department of Clinical Neuroscience, Karolinska Institutet & Stockholm Health Care Services, Stockholm County Council, Karolinska University Hospital, Stockholm, Sweden.

^*^These authors contributed equally to this work
^#^To whom correspondence should be addressed: Carl M Sellgren, Dept. of Physiology and Pharmacology, Biomedicum C5, Solnavägen 9, 171 65 Solna, Sweden. Phone: +46 (0) 70-212 72 87. Email: [carl.sellgren@ki.se](mailto:carl.sellgren@ki.se)

**Supplementary Methods**

*Ethics*

All individuals signed a written informed consent before participating in the study, as approved by the Institutional Review Board of Partners HealthCare (Boston, MA, USA) and the Regional Ethical Review Boards in Stockholm, Sweden. All relevant ethical regulations were followed when performing the study.

*Vero E6 cells*

Authenticated Vero E6 cells (ATCC-CRL-1586), testing negative for Mycoplasma, were obtained from Sigma Aldrich, and maintained in Dulbecco’s modified eagle medium (DMEM, Cytiva) supplemented with 5 % heat-inactivated fetal bovine serum (FBS, Cytiva), 100 U/mL penicillin and 100 μg/mL streptomycin (Cytiva).

*iPSC reprogramming*

Two healthy human iPSC lines (males) were obtained from the MGH Neurobank. Briefly, iPSC colonies were obtained using mRNA reprogramming in a feeder-free culture system as described previously (1). Stable iPSCs were expanded in mTESR plus media (STEMCELL Technologies) and on biolaminin 521 LN-coated (BioLamina) plates. iPSCs were then purified using MACS with anti-TRA-1–60 MicroBeads (Miltenyi Biotec) on LS columns. All fibroblasts and iPSCs were screened and found negative for Mycoplasma and stained positive for pluripotency markers like octamer-binding transcription factor 4 (POU domain, class 5, transcription factor 1) and TRA-1–60.

*Brain organoid cultures*

Undirected brain organoids were prepared from single cell suspension of human iPSCs as previously described (2). Briefly, embryoid bodies (EBs) were generated by seeding 9000 single cells in each well of a low attachment 96-well U-bottom plate with Y-27632 ROCK inhibitor (10 μM) for one day. EB media was replaced on day 5 with Neural induction media in the same 96-well plate. On day 10-11, EBs were embedded in 30μl Matrigel (Corning) using sheets of dimpled parafilm and incubated for 20 min at 37°C as previously detailed. Matrigel embedded single EBs were then either transferred to a 24-well plate in 3ml of expansion media per well, on an orbital shaker (90 rpm), or to a 12-well miniaturized bioreactor (largely overlapping with the design proposed by Qian et al.) (3), until further use.

*Induced microglia-like cells*

iMGs were derived from monocytes (donated from one healthy male) using established methods previously described in detail (1,4). Briefly, whole blood was collected into vacutainer cell preparation tubes containing sodium citrate as an anticoagulant (Becton, Dickinson and Company) and processed as per the manufacturer’s instructions. Peripheral blood mononuclear cells (PBMC) were isolated, washed twice with PBS by centrifugation and suspended in heat-inactivated fetal bovine serum (FBS; Sigma) containing 10% DMSO (Sigma). The cell suspension was then divided into aliquots and cryopreserved. Generation of induced microglia-like cells (iMG) from PBMCs were carried out using methods previously described (4), with minor modifications. Briefly, cryopreserved PBMC samples were transferred from liquid nitrogen freezer to a 37 °C water bath. Once the cell suspension had been thawed, it was gently pipetted into 10 ml of pre warmed complete RPMI medium (CM) consisting of basal RPMI-1640 supplemented with 10% heat-inactivated FBS and 1% penicillin-streptomycin (P/S; Thermo Fisher Scientific). Following centrifugation (300 *g* for 5 min at room temperature), the supernatant was discarded, and the cell pellet was resuspended in the appropriate volume of CM. Isolated PBMCs were cultured at a density of 5x10^5^ cells/1 ml CM on 24-well plates coated with Geltrex (Thermo Fisher Scientific).  After 24 h of incubation, the media was replaced with RPMI-1640 supplemented with 1x glutamax (Life Technologies), 1% P/S and 0.1 μg ml^−1^ of interleukin-34 (R&D Systems) and 0.01 μg ml^−1^of granulocyte macrophage colony-stimulating factor (GM-CSF; R&D Systems). After 7 days, fresh media was added. At day 11, cells were used for infection.

*Virus isolate*

A Wuhan variant of SARS-CoV-2 (GenBank: MT093571), originally obtained from the Public Health Agency of Sweden (<https://zenodo.org/record/4722502#.YmqVZC8RqMI>), was propagated on 90% confluent Vero E6 cells for 3 days at 37 °C. Cell debris were removed by centrifugation at 300 RCF for 5 minutes and the viral supernatant was aliquoted into cryovials and stored at -80 °C. Viral titers were quantified by PFU assay.

*Viral infection*

All experimental studies involving infectious SARS‐CoV-2 were performed within the biosafety level 3 (BSL3) facility at Karolinska Institute. From single cell dissociation experiments, approximately 1x10^6^ cells were found to be present in a D56 organoid. Based on this, 3x10^5^ PFU/ml of SARS-CoV2 was used to infect organoids for 2h (such that the estimated MOI was 1 for periphery and 0.3 for the whole organoid ) in a spinning bioreactor. Following viral exposure, the organoids were washed twice with PBS and transferred into fresh medium (2ml) to follow the course of infection. To monitor viral replication, 100μl of medium was collected at specified time points, centrifuged at 300g for 5 min and the supernatant was collected in trizol for qPCR analysis. For single cell RNA sequencing (scRNA-seq), the viral load was reduced to 1 x10^5^ PFU/ml (MOI 0.1) in order to reduce the percentage of cell death observed with the previous MOI.

*Plaque forming unit assay*

PFU assays was performed on 24-well cell culture plates seeded with 2x10^5^ Vero E6 cells per well. A serial dilution of the inoculum medium (consisting of either the viral stock or experimental samples in DMEM) was prepared and 200μL used to infect each well. The plates were then incubated for 1 hour at 37 °C, 5% CO2. After the incubation period, the wells were washed twice with PBS and 1 mL of overlay medium (2:3 mix of 3% high density carboxymethyl cellulose and complete DMEM medium) was added to each well and the plates incubated for 3 days at 37 °C, 5% CO2. The plates were subsequently inactivated with 1 mL of 10% formaldehyde solution overnight at RT, washed twice with PBS, stained with 200μL of crystal violet solution for 30 min at room temperature and the plaques counted.

*Immunohistochemistry*

Organoids were fixed with 4% paraformaldehyde for 20 min at RT followed by 24h in 30% sucrose in PBS. Organoids were then embedded in OCT (VWR) and frozen at –80^o^C. Cryosections (16 μm) of brain organoids were incubated with blocking solution for 1h (10% of Normal Donkey serum or 3% BSA in 0.3% Triton in PBS). Blocked cryosections were incubated with the respective primary antibodies, diluted in blocking solution and incubated overnight at 4°C:, anti-b-III-tubulin (mouse, Promega G712A, 1:500), Cleaved caspase-3 (rabbit, Cell Signaling, 1:300), anti-PAX6 (mouse, Developmental Studies Hybridoma Bank, 1:100), anti-SOX10 antibody (goat, R&D AF2864, 1:50), anti-OLIG2 (goat, R&D AF2418, 1:100), anti-GFAP (mouse, Sigma G3893, 1:100), anti-IBA1 (rabbit, Wako 019-19741, 1:100), anti-MMP14 (mouse, R&D MAB918-SP, 1:100 ), anti-ISG15 (mouse, Santa Cruz Ltd sc166755, 1:100), anti-PSD95 (mouse, Abcam 13552, 1:100), anti-PDGFRB (goat, R&D AF385-SP, 1:200), anti-LAMP2 (goat, R&D AF6228-SP, 1:200), SARS-CoV-2 (2019-nCoV) Nucleoprotein / NP Antibody (rabbit, Nordicbiosite 158-40143, 1:300) and anti-dsRNA (clone 9D5) (mouse, absolute antibody, 1:200) that previously has been validated against several structural proteins of SARS-CoV-2 (5). All used antibodies were checked regarding sensitivity and specificity using previous literature as well as in-house testing. Cryosections were washed three times with 1xPBS and incubated with the secondary antibody for 1h at RT. Secondary antibodies (conjugated to Alexa Fluor 488, 555 and 647) were purchased from Life Technologies and used at a 1:500 dilution. Then, samples were washed three times with 1xPBS, incubated for 5 minutes with fluorescent nuclear DAPI stain (VWR; 1:500), and mounted with DAKO immunofluorescence mounting media (Life Technologies). Image acquisition was performed using a Zeiss LSM800 confocal microscope (40X/1.2 W objective). Super resolution imaging acquisition was performed using a Zeiss LSM900-Airy2 scan confocal microscope (63X/1.4 oil objective) for representative images.

*Image quantification*

Immunofluorescence images were acquired using Zeiss LSM-800 confocal, LSM900-Airy*2* (super-resolution) microscope and analyzed using CellProfiler software to measure and classify cells according to the expression of the selected markers. First, the software was trained to automatically segment the images into: (1) cells (DAPI^+^ objects); (2) NP or casp3 objects, followed by relating objects (1) and (2). “mid” indicates the region of interest defined around the organoid core and “edge” refers to the region of interest at the organoid periphery where most of viral NP signal was detected. For cell type specific quantifications, (2) was related to (3) objects segmented by cell type specific markers stained by antibodies indicated above. Finally, the related objects were expressed as a percentage of DAPI^+^ cells. For validation of DE genes, 40X confocal images were obtained as tiles for the whole organoid and were processed in ImageJ/FIJI software (v2.1) for colocalization analyses.

For quantification of microglial engulfment of post-synaptic termini, images were acquired throughout the organoid while focusing on areas with IBA1^+^ cells (blinded to condition). The images were then processed using ImageJ/FIJI software (v2.1) macro consisting of the following steps: duplication of the red channel (corresponding to IBA1); background subtraction (rollingball set to 20); conversion to binary image; filters set to maximum and minimum of radius=5 followed by particles regarded as ROIs (Region of Interest) with size >1000 pixels. Then, for each ROI in a FOV (field of view), the green channel (corresponding to PSD-95) was used to set threshold (50, 255) and particles with size >3 pixels (circularity 0.01-1.00) were analyzed for number and area of each synaptic puncta. Total synaptic area in a ROI was normalized by the area of the corresponding ROI to get a measure of engulfment. The same set of images were used to measure area of colocalization between channels corresponding to MAP2 and PSD-95, respectively, followed by normalization with total MAP2 area in a FOV. For microglial morphology, representative images were processed in Imaris software (v9.1) and 3D contour surfaces were generated upon segmentation. For quantification, Z-stacks were processed, and maximum intensity projections were thresholded to create a binary mask. Shape descriptor parameters such as perimeter, circularity index and Feret’s diameter of IBA1^+^ cells were measured in ImageJ/ FIJI.

*Quantitative PCR (qPCR)*

RNA extraction was performed using DirectZol RNA-Miniprep Kit (Zymo Research Inc.), following the manufacturer’s protocol. Samples collected in TRI reagent were applied to Zymo-spin columns. DNase I treatment was performed for samples with genomic DNA. Bound RNA was washed, diluted in 30μl nuclease-free water and stored at -80°C. Quality and concentration of extracted RNA was determined using a NanoDrop (Thermofischer Scientific). Total RNA was reverse-transcribed to cDNA using the High-Capacity RNA-to-cDNA Synthesis kit (Thermofischer Scientific) following manufacturer’s protocol. Reverse transcription reactions were carried out in 20μl reaction volume containing 1μg of input RNA with the following thermal cycler conditions: 37°C (60 minutes) + 95°C (5 minutes) + 4°C (∞). cDNA was further diluted to 1:3 and used as templates for PCR reactions.

PCR reactions were performed using the StepOnePlus™ Real-time PCR system (Applied Biosystems, Thermofischer Scientific) with PowerTrack™ SYBR Green Master Mix. Expression of viral mRNA was assessed by evaluating threshold cycle (Ct) values. Relative expression levels of N gene in cell lysates were normalized against the housekeeping genes GAPDH (glyceraldehyde-3-phosphate dehydrogenase) and Actin Beta (*ACTB*), according to the Delta delta Ct method. Absolute quantification of viral gene expression levels in the supernatant were performed using the standard curve method. Primer sequences used in this study are:

GAPDH Forward – GGTGGTCTCCTCTGACTTCAACA

GAPDH Reverse – GTGGTCGTTGAGGGCAATG

ACTB Forward – CACCAACTGGGACGACAT

ACTB Reverse – ACAGCCTGGATAGCAACG

N gene Forward – CATTGGCATGGAAGTCACAC

N gene Reverse – TCTGCGGTAAGGCTTGAGTT

RdRp Forward – CGCATACAGTCTTRCAGGCT

RdRp Reverse – GTGTGATGTTGAWATGACATGGTC

*Dissociations for single cell RNA sequencing*

Briefly, three organoids per condition (control, 24hpi, 72hpi) were washed twice with DPBS without ions and cut into small pieces using a sterile scalpel. Single cell suspension was generated using neural dissociation kit (Miltenyi Biotec) according to manufacturer’s protocol. Microglia was enriched using CD11b magnetic microbeads (Miltentyi Biotec) using MACS. Viability and cell numbers were assessed for both MACS-enriched and flow-through fractions before proceeding with the 10X protocol.

*Single-cell RNA-sequencing*

Two single cell suspensions per condition were loaded onto a single Chromium controller chip v3.1 (10X Genomics), with a target output of 6000-7000 cells per channel. For the six loaded channels, GEM generation, barcoding, cDNA amplification and library preparation was performed using the Single-cell 3’ Gel Bead and Library v3.1 kit (10X Genomics) at the Eukaryotic Single Cell Genomics facility (SciLifeLab, Sweden). Amplified cDNA and final libraries were evaluated on a Bioanalyzer for quality control and sequenced together on Illumina NovaSeq 6000 platform.

*Data processing*

Sequenced data was processed through the CellRanger Software (5.0 ,10X Genomics) and transcripts were aligned to a combined reference of Human GRCh38-3.0.0 and SARS-Cov-2 genome (GenBank: MT093571.1). Exonic-read mapping-based feature-barcode matrices from CellRanger were further subjected to quality control measures where low-quality cells with < 200 uniquely expressed genes and high percentage of mitochondrial reads (>20%) were filtered out. Doublets were identified using scDblFinder package (6) and removed with caution. Genes expressed in fewer than 5 cells were filtered out. Filtered count matrices were merged and analyzed downstream using the Seurat package (7). Expression data per experimental condition was normalized using the regularized negative binomial regression method implemented in SCTransform (8), while regressing out the difference between the G2M and S phase scores, which served as a confounding factor in our dataset. Anchors across conditions were identified using FindIntegrationAnchors function and samples were integrated using canonical correlation analysis (CCA) implemented in the IntegrateData function of Seurat. Linear data compression using principal component analysis (PCA) was performed on 3000 highly variable genes. Top 30 PCs were used as the input to perform non-linear dimensionality reduction using UMAP.

*Clustering and cell-type identification*

We constructed a k-nearest neighbor (KNN) graph based on Euclidean distance in 30 PCs and performed unsupervised graph-based clustering using the Louvain algorithm (modularity resolution = 0.8) with functions provided in Seurat. Preliminary clustering of 39,808 cells identified 21 clusters yielding a clear separation of neuro-ectodermal clusters. Non-neuroectodermal clusters that did not express known marker genes related to cell types present in the brain, were removed. The remaining 26,148 cells were then subjected to second-level clustering in an integrated space (resolution= 0.6), yielding 16 final clusters. The clustering was visualized using UMAP embedding in two dimensions. Top differentially expressed genes conserved across conditions for the final clusters were identified using a non-parametric Wilcoxon rank-sum test in FindConservedMarkers function. P-values were adjusted based on Bonferroni correction and genes with at least 25% cluster-specific expression, >0.25 average log-fold change, and FDR<0.01 were chosen to identify the clusters. All cells were assigned scores based on cell type-specific gene modules to further identify broad cell types such as neurons, astrocytes, microglia and oligodendrocytes. A priori set of markers curated based on previous studies of cerebral organoids and developing fetal brain was explored to manually annotate the clusters. Additionally, we leveraged existing published fetal brain and organoid scRNA-seq datasets and undertook a correlation-based method (9) (Spearman) to compare our annotated clusters to transcriptional profiles of previously annotated cell types.

*Differential expression testing and functional interpretation*

We performed differential gene expression analyses on each cluster across conditions on log-normalized values using the MAST package (10) in R. DEGs were considered significant if their Benjamini-Hochberg-adjusted P-value was < 0.05 and a log2 fold change was > 0.25. A pseudo-bulk differential analysis was performed for microglia using DESeq2 with likelihood ratio testing. Significant DEGs were used to perform GO term overrepresentation analysis using the enrichR package. Gene set enrichment analysis (GSEA) was performed on ranked DEGs using fgsea package (11) for pathway analysis (KEGG, GO:BPdatabase). Gene sets were limited by minSize=3 and nPerm=10000. Normalized enrichment scores were calculated and plotted for pathways with adjusted p-values < 0.05. Statistical significance of association of DEGs with gene signatures from published datasets was performed using Fischer’s exact test to obtain a p-value (<0.05, adjusted with BH method) and Odds ratio (OR). Number of genes expressed in the respective cell type was used as the genomic background.

*Intercellular interaction*

Cellular crosstalk was inferred via ligand-receptor pair expression using CellphoneDB package(v2) (12) in python. Statistical analysis was performed within the package with default parameters (1000 iterations) and no subsampling was done.

*Statistics*

No statistical methods were used to determine needed sample sizes. The assumptions of each used statistical test were checked. Data was analyzed using R for Mac OS (version 4.2.1) or Prism 9 for macOS (version 9.3.1). All reported p-values are two sided and type of statistical test is reported in the figure legends or in the main text.

**Supplementary Results**

*Confirming the expression of SARS-CoV-2 entry factors*

As described in the main text, we assessed the expression of previously identified entry factors for SARS-CoV-2 in the generated single cell data and observed that the overall basal RNA expression for most factors was relatively low (**Supplementary** **Fig. 5g**). However, to some extent, we may have at least partly underestimated the expression of these entry factors by enriching for viable cells displaying low tropism and low expression of entry factors, or not thoroughly capturing the expression at the present sequencing depth. For *ACE2*, we then also confirmed expression by IHC (**Supplementary Fig. 5h**). In line with previous reports, cells belonging to the choroid plexus showed relatively higher *ACE2* expression (13,14), and we also observed a more pronounced expression in a subset of neurons with midbrain dopaminergic markers (**Supplementary Fig. 5g-i).** However, this subset of neurons did not exhibit any differential response in relation to other neuronal clusters across conditions (**Supplementary table 4)**. Similar to *ACE2* expression, proteases involved in viral S protein priming (15) (*TMPRSS2* and *TMPRSS4*) also displayed low expression, whereas *FURIN* (16) and infection potentiating factor (Neuropilin 1: *NRP1*,) had a broader cell type distribution that included astrocytes, perivascular cells, endothelial cells and microglia (**Supplementary Fig. 5g**). Microglia also expressed other predicted entry factors (*CTSL* and *CTSB,* Cathepsin L and B*)*, purported to act as substitutes for TMPRSS2 (15) (**Supplementary Fig. 5g**).

*Metabolic dysregulation across cell types*

As described in the main text, upon exposure to SARS-CoV-2, our CNS cell types underwent major stress that results in metabolic changes which are likely to impact cellular communication such as the important interplay between astrocytes and neurons. As SARS-CoV-2 then also relies on excessive glucose for its replication (17), and shunts glucose from glycolytic pathways to fuel oxidative phosphorylation and ATP production in a process known as the Warburg effect (18), we specifically studied pathways pertaining to cellular respiration. In neurons (24hpi), we also observed enrichment in pathways for oxidative phosphorylation, electron transport chain coupled to ATP synthesis, while those of glucose and pyruvate metabolism were downregulated (**Supplementary Table 4)**. Furthermore, transcription of glucose transporter, GLUT1 (*SLC2A1*), lactate dehydrogenase (*LDHA*) along with monocarboxylate transporter, MCT4 (*SLC16A3*), was downregulated in astrocytes at 24 and 72hpi, indicating deficits in glucose export and redox cycling necessary for neuronal energization (19) (**Supplementary Table 4)**.

**Supplementary Figure Legends**

**Supplementary Figure 1.** Representative confocal images (40X) showing markers corresponding to **(a)-(b)** progenitors PAX6, KI67, SOX2 **(c)-(d)** mature astrocytes GFAP, AQP4 in 56 DIV and 130 DIV organoids, respectively. Scale bars in representative images: 20um **(e)** Confocal images (40X) showing co-expression of TMEM119 in a subset of IBA1 and CD68-expressing microglia in 130 DIV organoids. Arrows indicate TMEM119-positive and -negative microglial cells. Scale bars in representative images: 10um. Nuclei is stained with DAPI in all confocal images. **(f)** qPCR analysis on 130 DIV organoid cell lysates from two lines showing relative mRNA expression of microglial markers *AIF1*, *PU.1*, *P2RY12* and *TMEM119.* Center values represent means and error bars indicate S.E.M.

**Supplementary Figure 2. (a)** qPCR analysis of SARS-CoV-2 infected cerebral organoids at 56 DIV (n=3; squares and triangles) and VeroE6 cells (circles) showing log fold changes in viral transcripts corresponding to nucleocapsid gene (*N* for organoids and VeroE6 cells) and the RNA-dependent RNA polymerase gene (*Rdrp*; organoids). A repeated 2-way ANOVA followed by posthoc tests revealed a significant increase in *N* gene expression for both VeroE6 cells (48hpi vs 6 hpi; *P*=0.027) and organoids (72hpi vs. 6hpi; *P*=0.015). For *Rdrp* expression, we also observed an increased expression in organoids comparing the 48hpi and the 6hpi time points *(P=*0.007) as well as by comparing the 72hpi and 6hpi time points (*P*=0.015). For between groups comparisons (VeroE6 cells vs. organoids) of *N* gene expression, we observed an increased N gene expression in veroE6 cells at 6hpi *(P=*0.024) and 24hpi *(P=*0.037).  **(b)** NP-positive and CASP3-positive cells expressed as percentage of DAPI from two 56 DIV infected organoids (organoids indicated by different shapes in graph). Each data point is an average of 2-3 FOVs (field of views) per section. “mid” indicates region of interest around the organoid core and “edge” refers to the region of interest at organoid periphery where most of viral NP signal was detected. **(c)** Bar plot showing distribution of CASP3-positive cells amongst different cell-type markers, expressed as percentage of DAPI from two 56 DIV infected organoids (represented as triangles and circles). Mean represents an average of 4 FOVs representing different areas on the same section. **(d)** Confocal images (40X) showing colocalization of CASP3 with neuronal- (MAP2 and PAX6) and glial- (GFAP and SOX10) lineage markers. Scale bars in representative images: 20um **(e)** qRT-PCR on cell lysate fraction obtained following MACS with CD11b beads, showing expression levels of two microglial specific genes (*AIF1*, *TMEM119*). **(f)** Induced microglia (iMG) from a healthy donor, infected with SARS-CoV-2 (MOI 0.01) and stained for dsRNA (clone 9D5, for viral presence) and CASP3 (red), at 24hpi and **(g)** corresponding quantifications of dsRNA^+^ (green circle) and CASP3^+^ (black triangle) cells expressed as percentage of IBA1 expressing iMGs at two time points following infection. Three FOVs were acquired per replicate well and the counts expressed as a datapoint. Center values in each graph represent means and error bars represent S.E.M. All reported p-values are two-sided. **P*<0.05, ***P*<0.01.

**Supplementary Figure 3.** (a) Quantification of % Iba1^+^ cells in mock treated organoids and in SARS-CoV-2 infected organoids 72 hpi. (b) Quantification of % CD68^+^ cells in mock treated organoids and in SARS-CoV-2 infected organoids 72 hpi. **(c)** Clustering microglia cells displaying a less ramified morphology in the viral exposure model. (**d**) Microglia cell in the mock infected model with a more complex morphology. **(e)** Whole cell perimeter quantifications in microglia from mock and SARS-CoV-2 infected models. **(f)** Quantification of microglia circularity scores in mock and SARS-CoV-2 infected models. **(g)** Quantification of Feret’s diameter in microglia from mock and SARS-CoV-2 infected models. Datapoints in all graphs represent FOVs, data were analyzed using Mann-Whitney *U* tests. All reported p-values are two-sided. Images in (c) – (d) were created using Imaris and schematics in (c) – (e) were created using Biorender.

**Supplementary Figure 4. (a)** Bar plot showing number of cells obtained per condition post quality control (see methods). **(b)** Scatter plot of percentage of mitochondrial reads versus number of expressed genes of the entire single-cell dataset prior to quality control. Red line indicated the applied cutoff for mitochondrial percentage. Violin plots showing the distribution of transcript counts (left) and expressed genes per cell (right) across 6 samples on a **(c)** linear scale and **(d)** log scale [Mock control- Lane 1&2; 24hpi- Lane 3&4; 72hpi- Lane5&6]. **(e)** Violin plots showing the distribution of transcript counts (top) and expressed genes per cell (bottom) across every identified cell type cluster. Center values represent median. **(g)** UMAP plots of overall embedding of the dataset integrated with two tools, CCA (left) and Harmony (right) as validation confirming no method-specific bias. Each cell is colored by its sample showing uniform distribution and no batch effects in the clustering analysis. **(h)** Bar plot with percentage cell type composition across individual samples. **(i)** UMAP plots displaying the distribution of QC metrics, percentage of mitochondrial reads (left), and calculated doublet score (right) per cell across all clusters.

**Supplementary Figure 5.** Cell type identification using a developmental single-cell dataset, *Pollen et al.,* 2019 (20) (human primary and human organoids), as a reference dataset. Expression of top marker genes for each reference cell type (labelled on the left) are visualized across all cells on standard UMAP embeddings of our dataset. Cells are colored according to the estimated joint density of multiple marker genes represented by the scale on the right of each plot.

**Supplementary Figure 6.** **(a)** UMAP plots showing the expression of a canonical marker gene per cellular group across all cells. Spearman-ranked correlation of cerebral organoid clusters (rows) to reference transcriptomes (columns) of **(b)** human primary fetal cortex (*Nowakowski et al*., 2017) (21), **(c)** integrated human cerebral organoid datasets (*Tanaka et al.*, 2020) (22), **(d)** human primary developmental dataset (*Pollen et al.,* 2015) (23) and **(e)** human primary developmental dataset (*Bhaduri et al.*, 2019) (24). **(f)** Dendrogram (left) and UMAP plot (right) integration space of microglial transcriptomic profiles comparing organoid-grown microglia to fetal (*Nowakowski et al*., 2017 & *Zhong et al., 2018*) and adult *(Hodge et al., 2019)* microglia. Cells are colored by their dataset. **(g)** Heatmap showing relative mRNA expression levels of proposed cellular entry factors utilized by SARS-CoV-2 virus across cell types. **(h)** Confocal images (40X) of 56 DIV organoid showing ACE2 expression (red) in B-III tubulin^+^ neurons (green). **(i)** UMAP plots highlighting a subset of neurons with *ACE2* expression along with expression of midbrain dopaminergic markers. This subset did not exhibit a differential response to SARS-CoV-2 in comparison to other neuronal clusters across clusters (Supplementary table 3). Consistent with an underestimation of *ACE2* expression due to e.g., negative selection based on viability, we observed more robust and heterogenous neuronal ACE2 staining using IHC (**Supplementary Fig. 5h**).

**Supplementary Figure 7.** Bar plots showing top significant pathways enriched for gene module A **(a)** and gene module B **(b)** found in microglia by over-representation analysis (BH-adjusted p<0.05). **(c)** Quantification of IHC staining corresponding to Fig. 6c of ISG15^+^ and IBA1^+^ cells (Mann-Whitney *U* test, *P*<0.0001). Center value represent median and error bars represent interquartile range. **(d)** UMAP plot showing sub clustering analysis of microglial cells colored by their identified microglial subclusters (top) and condition (bottom). No differentially expressed genes (log2FC>0.2; Benjamini Hochberg-corrected p-value<0.05) were found between the microglial subclusters**. (e)** Heatmap of differentially expressed genes in microglia upon SARS-CoV-2 infection using MAST (see Methods). **(f)** Top significant upregulated and down-regulated KEGG pathways between SARS-CoV-2 exposed microglia (24hpi+72hpi) versus mock-treated microglia obtained by GSEA (Benjamini Hochberg-corrected *P*<0.05; see Methods). Color bar shows Normalized expression score (NES). **(g)** Bar plots showing top pathways enriching for genes intersecting between IRM state and microglia from 72hpi observed in Fig. 6e. Dotted line indicates significance (Benjamini Hochberg-corrected *P<*0.05). **(h)** Violin plots showing normalized expression of microglial markers associated with disease and homeostasis across conditions. **(i)** Dot plot displaying expression of canonical ‘don’t-eat-me' signals in neuronal clusters across infected conditions. Size of the dot indicated fraction of cells expressing the gene. Scale bar indicated scaled-average expression values. All reported p-values are two-sided. **P*<0.0001.

**Supplementary Figure 8**. **(a)** UMAP embedding of astrocyte subclusters displaying the distribution of QC metrics such as number of reads (nCount_RNA), number of expressed genes (nFeature_RNA), and percentage of mitochondrial reads (percent. mito) per cell. **(b)** UMAP plot showing astrocyte subclusters with each cell colored by the sample. **(c)** Astrocytic subclusters are visualized as columns on the heatmap with relative expression levels of top differentially expressed marker genes (rows) of individual subclusters (using MAST test implemented in Seurat, adjusted p-value<0.05). Top bar is colored by each astrocyte subcluster. High expression values shown in yellow while low expression values shown in purple. **(d)** Dot plot showing average-scaled expression of genes related to astrocyte activation and function across astrocyte subclusters. **(e)** Violin plots of cells scored by A1-reactive and A2-reactive signatures as defined in Barbar et al., 2020 (25), across astrocyte subclusters. **(f)** UMAP plot showing the integrated embedding astrocytes and human iPSC-derived A1-astrocytes, as seen in Fig. 7d, colored by astrocyte subclusters.

**Supplementary Figure 9. (a)** UMAP embedding of choroid plexus subclusters displaying the distribution of QC metrics such as number of reads (nCount_RNA), number of expressed genes (nFeature_RNA), percentage of mitochondrial reads (percent.mito), and calculated G2M per cell. **(b)** UMAP plot showing choroid plexus subclusters with each cell colored by the sample. **(c)** Heatmap showing scaled expression of top differentially expressed markers by choroid plexus subclusters (obtained using MAST test implemented in Seurat, Benjamini Hochberg corrected p-value<0.05). Top bar is colored by each choroid plexus subcluster. High expression are values shown in yellow while low expression values in purple. Genes associated with identified mechanisms linked to SARS-CoV-2 exposure are highlighted in boxes.

**Supplementary Table Legends**

**Supplementary Table 1.** Differentially expressed genes between clusters to identify celltype specific markers conserved across all three conditions.

**Supplementary Table 2**. Percentage of total infected and bystander cells in control and infected conditions, along with infected cell types identified in each cluster.

**Supplementary Table 3.** Unbiased hierarchical clustering of differentially expressed genes across all three experimental conditions.

**Supplementary Table 4**. Pathway enrichment analysis for major cell types between experimental conditions.

**References**

1. Sellgren CM, Gracias J, Watmuff B, Biag JD, Thanos JM, Whittredge PB, et al. Increased synapse elimination by microglia in schizophrenia patient-derived models of synaptic pruning. Nat Neurosci. 2019 Mar 4;22(3):374–85.

2. Ormel PR, Vieira de Sá R, van Bodegraven EJ, Karst H, Harschnitz O, Sneeboer MAM, et al. Microglia innately develop within cerebral organoids. Nat Commun. 2018;9(1):4167.

3. Qian X, Jacob F, Song MM, Nguyen HN, Song H, Ming GL. Generation of human brain region–specific organoids using a miniaturized spinning bioreactor. Nat Protoc. 2018;13(3):565–80.

4. Sellgren CM, Sheridan SD, Gracias J, Xuan D, Fu T, Perlis RH. Patient-specific models of microglia-mediated engulfment of synapses and neural progenitors. Mol Psychiatry. 2017 Feb 1;22(2):170–7.

5. Scherer KM, Mascheroni L, Carnell GW, Wunderlich LCS, Makarchuk S, Brockhoff M, et al. SARS-CoV-2 nucleocapsid protein adheres to replication organelles before viral assembly at the Golgi/ERGIC and lysosome-mediated egress. Sci Adv. 2022 Jan 7;8(1):eabl4895.

6. Germain PL, Lun A, Macnair W, Robinson MD. Doublet identification in single-cell sequencing data using scDblFinder. F1000Res. 2021 Sep 28;10.

7. Stuart T, Butler A, Hoffman P, Hafemeister C, Papalexi E, Mauck WM, et al. Comprehensive Integration of Single-Cell Data. Cell. 2019 Jun;177(7).

8. Hafemeister C, Satija R. Normalization and variance stabilization of single-cell RNA-seq data using regularized negative binomial regression. Genome Biol. 2019 Dec 23;20(1).

9. Fu R, Gillen AE, Sheridan RM, Tian C, Daya M, Hao Y, et al. clustifyr: an R package for automated single-cell RNA sequencing cluster classification. F1000Res. 2020 Jul 16;9.

10. Finak G, McDavid A, Yajima M, Deng J, Gersuk V, Shalek AK, et al. MAST: a flexible statistical framework for assessing transcriptional changes and characterizing heterogeneity in single-cell RNA sequencing data. Genome Biol. 2015 Dec 10;16(1).

11. Sergushichev A. An algorithm for fast preranked gene set enrichment analysis using cumulative statistic calculation. 2016;

12. Efremova M, Vento-Tormo M, Teichmann SA, Vento-Tormo R. CellPhoneDB: inferring cell–cell communication from combined expression of multi-subunit ligand–receptor complexes. Nat Protoc. 2020 Apr 26;15(4).

13. Chen R, Wang K, Yu J, Howard D, French L, Chen Z, et al. The Spatial and Cell-Type Distribution of SARS-CoV-2 Receptor ACE2 in the Human and Mouse Brains. Front Neurol. 2020;11:573095.

14. Pellegrini L, Albecka A, Mallery DL, Kellner MJ, Paul D, Carter AP, et al. SARS-CoV-2 Infects the Brain Choroid Plexus and Disrupts the Blood-CSF Barrier in Human Brain Organoids. Cell Stem Cell. 2020;27(6):951-961.e5.

15. Hoffmann M, Kleine-Weber H, Schroeder S, Krüger N, Herrler T, Erichsen S, et al. SARS-CoV-2 Cell Entry Depends on ACE2 and TMPRSS2 and Is Blocked by a Clinically Proven Protease Inhibitor. Cell. 2020;181(2):271-280.e8.

16. Johnson BA, Xie X, Bailey AL, Kalveram B, Lokugamage KG, Muruato A, et al. Loss of furin cleavage site attenuates SARS-CoV-2 pathogenesis. Nature. 2021 Mar 11;591(7849):293–9.

17. Ana Campos Codo A, Gastã Davanzo G, Campos Codo A, de Brito Monteiro L, Fabiano de Souza G, fanie Primon Muraro S, et al. Elevated Glucose Levels Favor SARS-CoV-2 Infection and Monocyte Response through a HIF-1a/ Glycolysis-Dependent Axis ll Elevated Glucose Levels Favor SARS-CoV-2 Infection and Monocyte Response through a HIF-1a/Glycolysis-Dependent Axis. Cell Metab. 2020;32:437-446.e5.

18. Stoolman JS, Chandel NS. Glucose Metabolism Linked to Antiviral Responses. Vol. 178, Cell. Cell Press; 2019. p. 10–1.

19. Weber B, Barros LF. The astrocyte: Powerhouse and recycling center. Cold Spring Harb Perspect Biol. 2015 Dec 1;7(12):20396–7.

20. Pollen AA, Bhaduri A, Andrews MG, Nowakowski TJ, Meyerson OS, Mostajo-Radji MA, et al. Establishing Cerebral Organoids as Models of Human-Specific Brain Evolution. Cell. 2019;176(4).

21. Nowakowski TJ, Bhaduri A, Pollen AA, Alvarado B, Mostajo-Radji MA, di Lullo E, et al. Spatiotemporal gene expression trajectories reveal developmental hierarchies of the human cortex. Science (1979). 2017;358(6368).

22. Tanaka Y, Cakir B, Xiang Y, Sullivan GJ, Park IH. Synthetic Analyses of Single-Cell Transcriptomes from Multiple Brain Organoids and Fetal Brain. Cell Rep. 2020;30(6).

23. Pollen AA, Nowakowski TJ, Chen J, Retallack H, Sandoval-Espinosa C, Nicholas CR, et al. Molecular Identity of Human Outer Radial Glia during Cortical Development. Cell. 2015;163(1).

24. Bhaduri A, Andrews MG, Mancia Leon W, Jung D, Shin D, Allen D, et al. Cell stress in cortical organoids impairs molecular subtype specification. Nature. 2020;578(7793):142–8.

25. Barbar L, Jain T, Zimmer M, Kruglikov I, Sadick JS, Wang M, et al. CD49f Is a Novel Marker of Functional and Reactive Human iPSC-Derived Astrocytes. Neuron. 2020;107(3):436-453.e12.
