# Supplementary figures and images for: SARS-CoV-2 promotes microglial synapse elimination in human brain organoids
Source: Mol Psychiatry. 2022 Oct 5;27(10):3939–50. doi: 10.1038/s41380-022-01786-2 (PMC9533278; doi:10.1038/s41380-022-01786-2)

**a**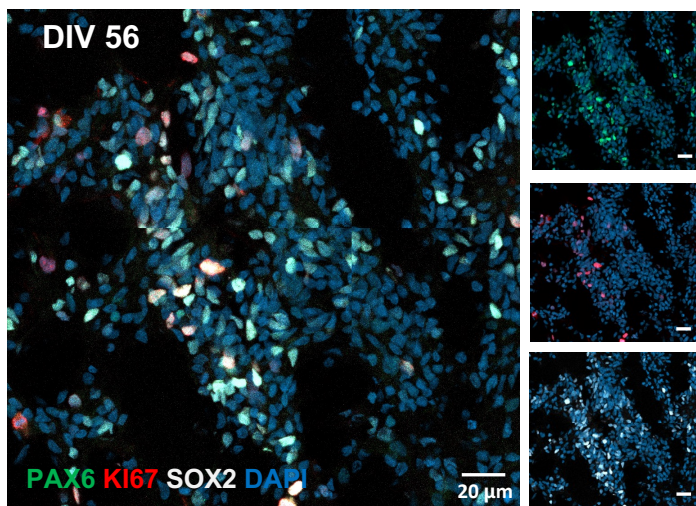**b**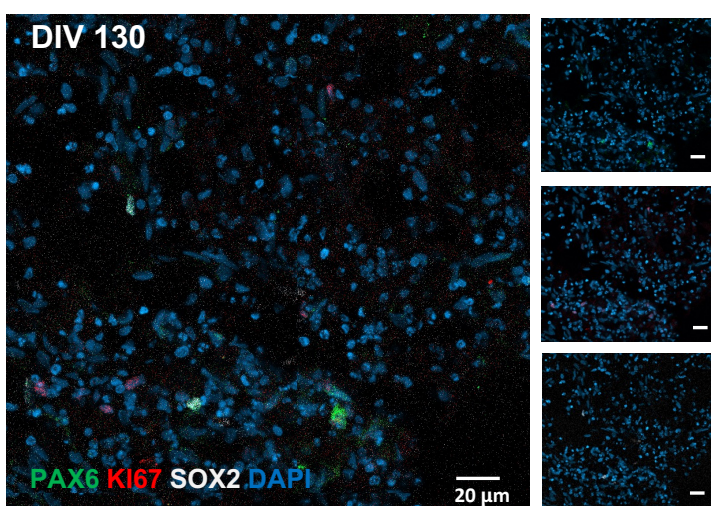**c**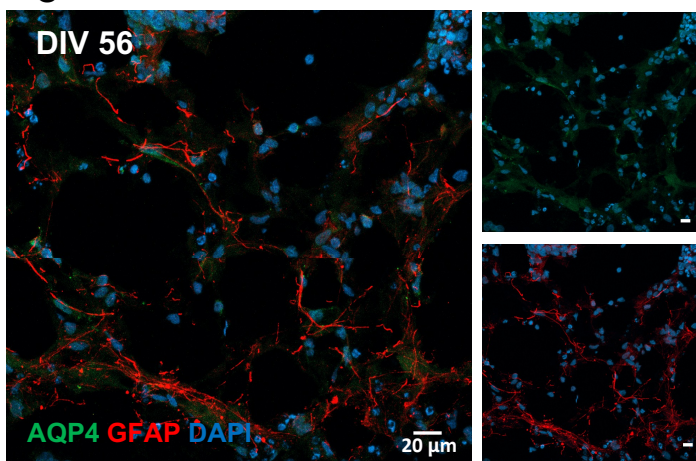**d**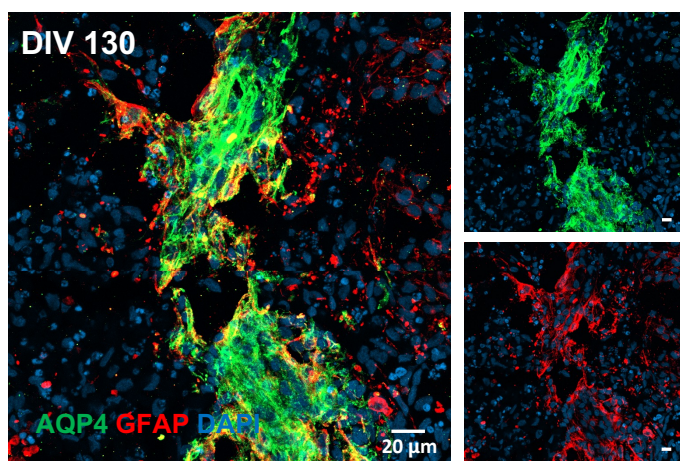**e****DIV 130**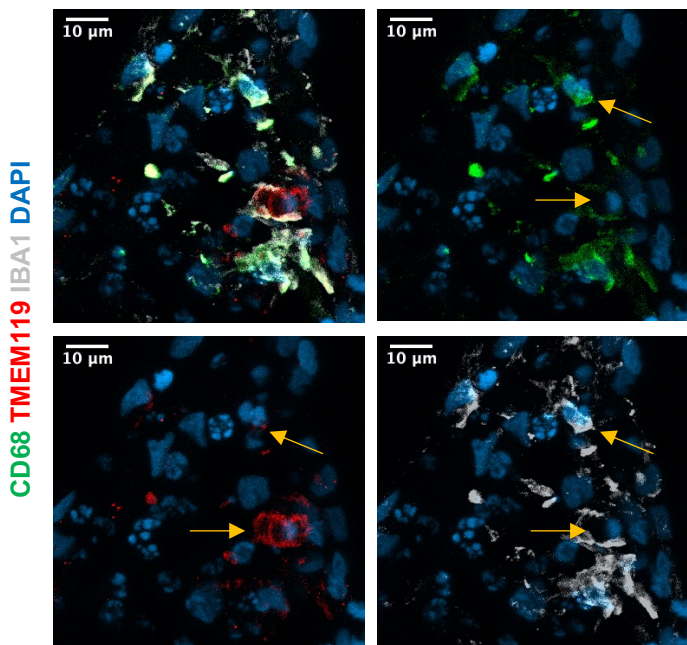**f****DIV 130**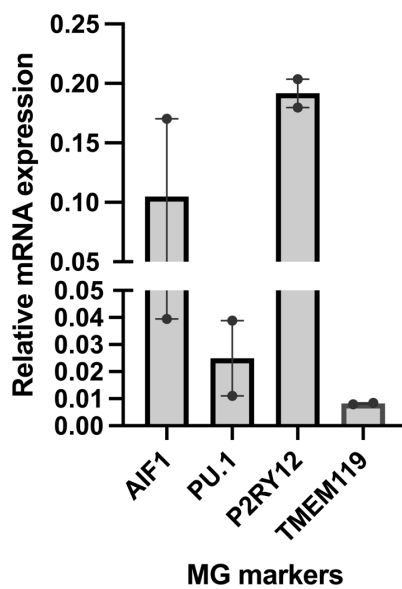

Supplement: Supplementary file 2 — Supplementary Figure 1 [file 41380_2022_1786_MOESM2_ESM.pdf]

**a**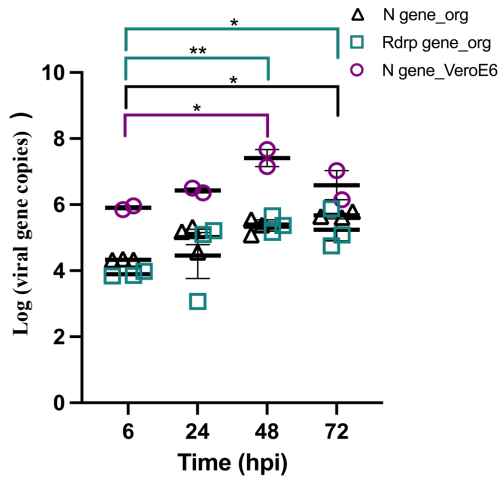**b**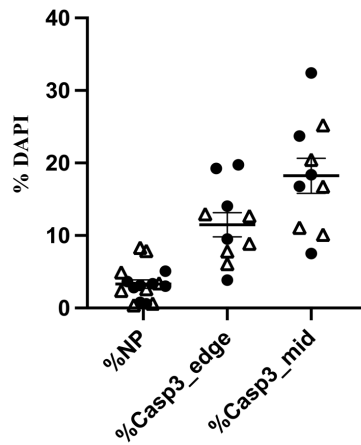**c**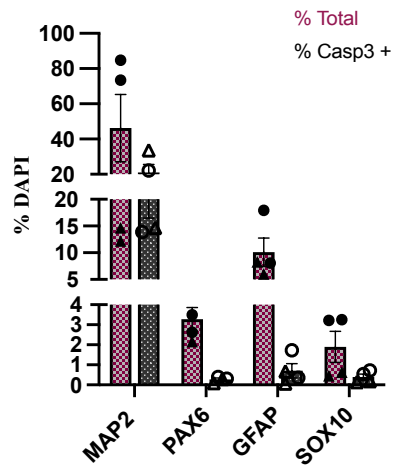**d**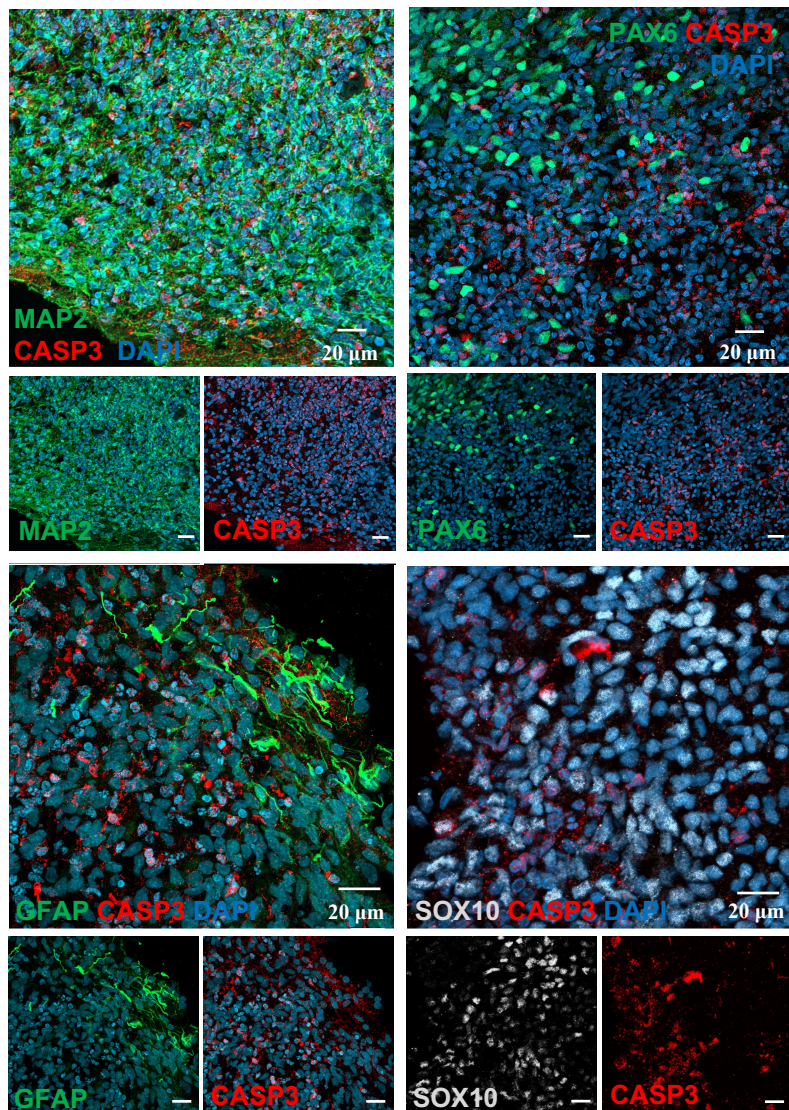**e**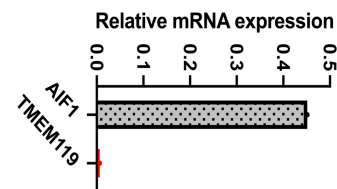**f**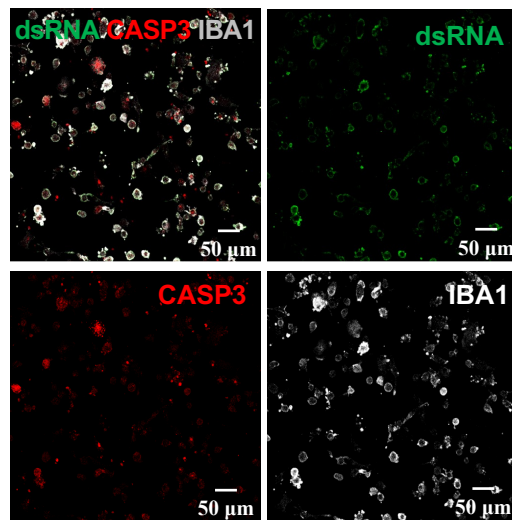**g**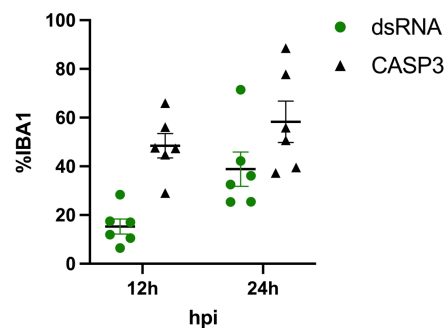

Supplement: Supplementary file 3 — Supplementary Figure 2 [file 41380_2022_1786_MOESM3_ESM.pdf]

**a.**

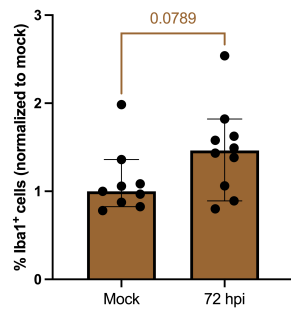

**b.**

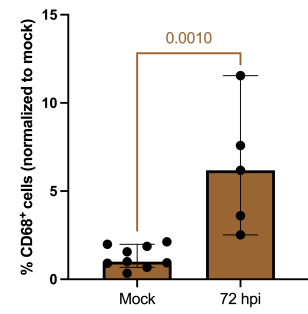

**c.**

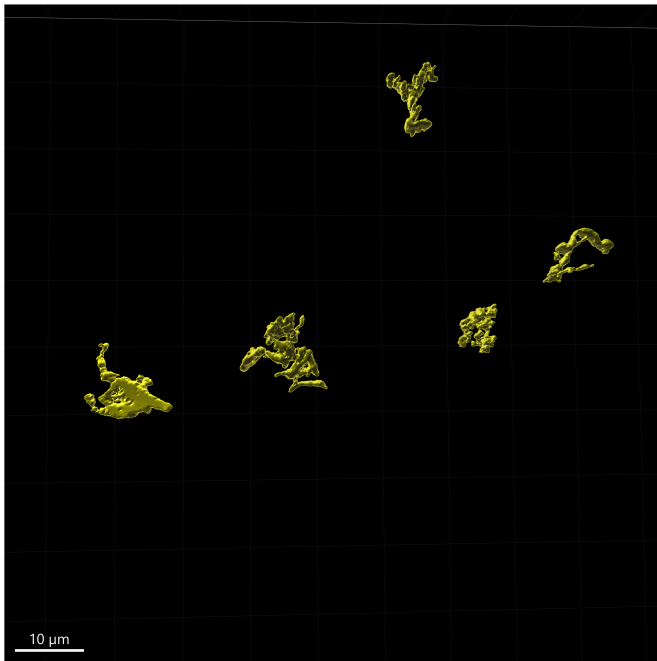

**d.**

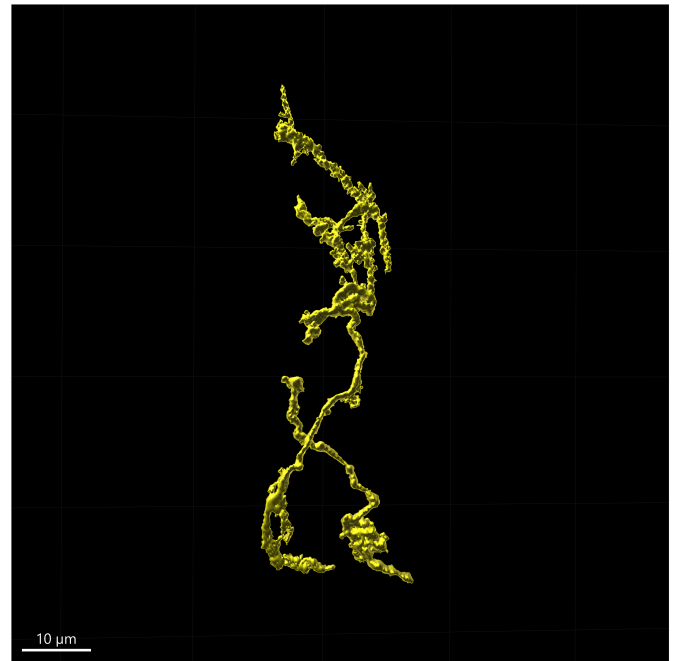

**e.**

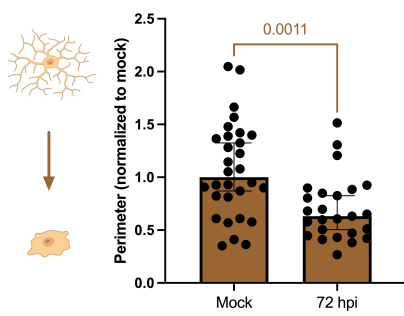

**f.**

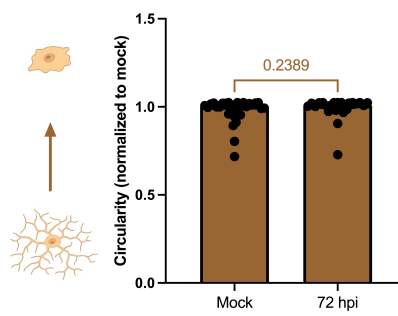

**g.**

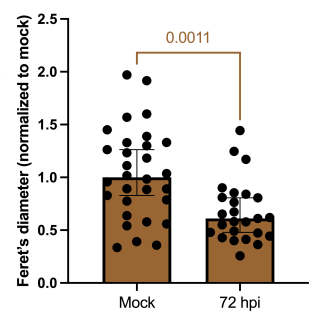

Supplement: Supplementary file 4 — Supplementary Figure 3 [file 41380_2022_1786_MOESM4_ESM.pdf]

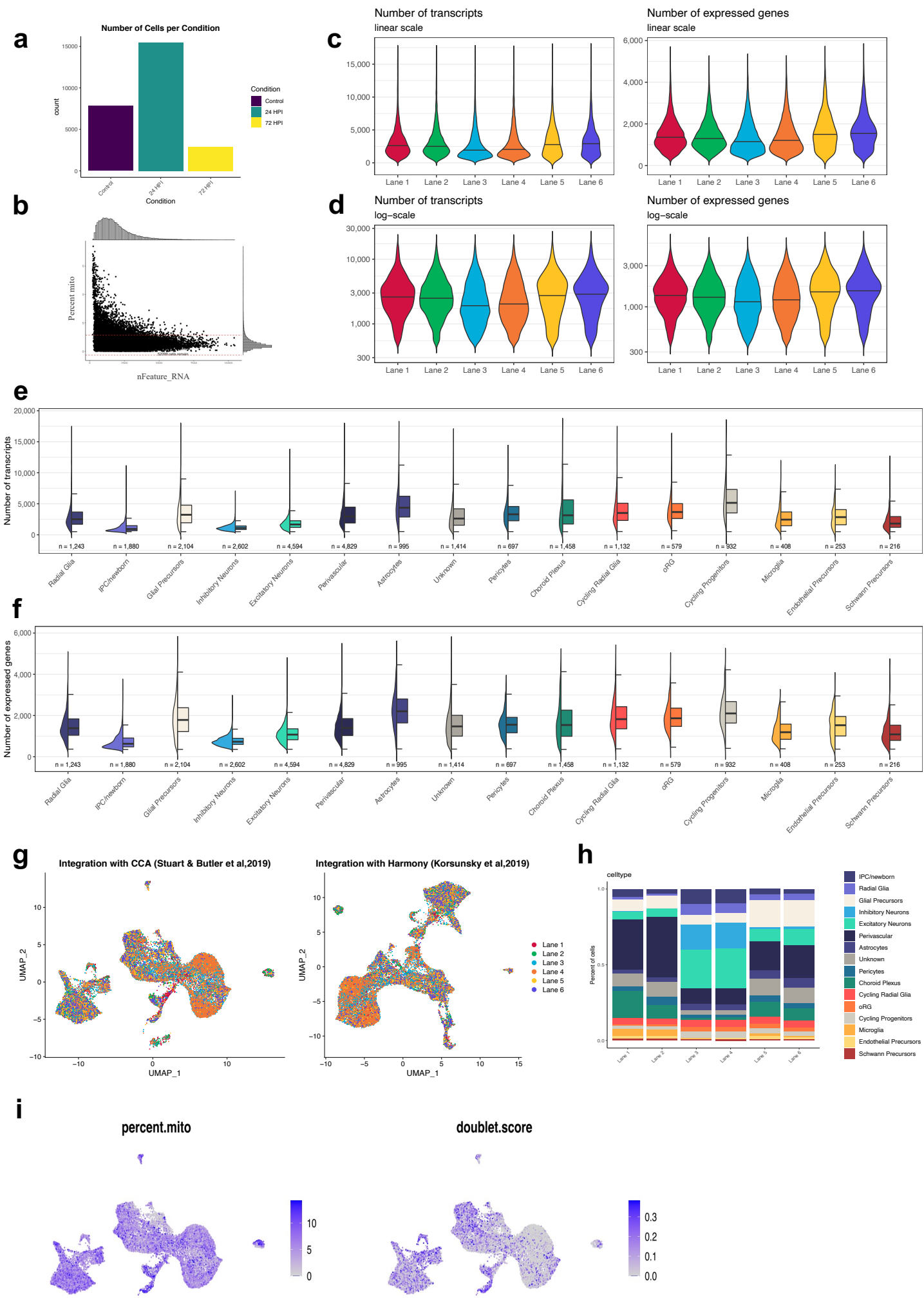

Supplement: Supplementary file 5 — Supplementary Figure 4 [file 41380_2022_1786_MOESM5_ESM.pdf]

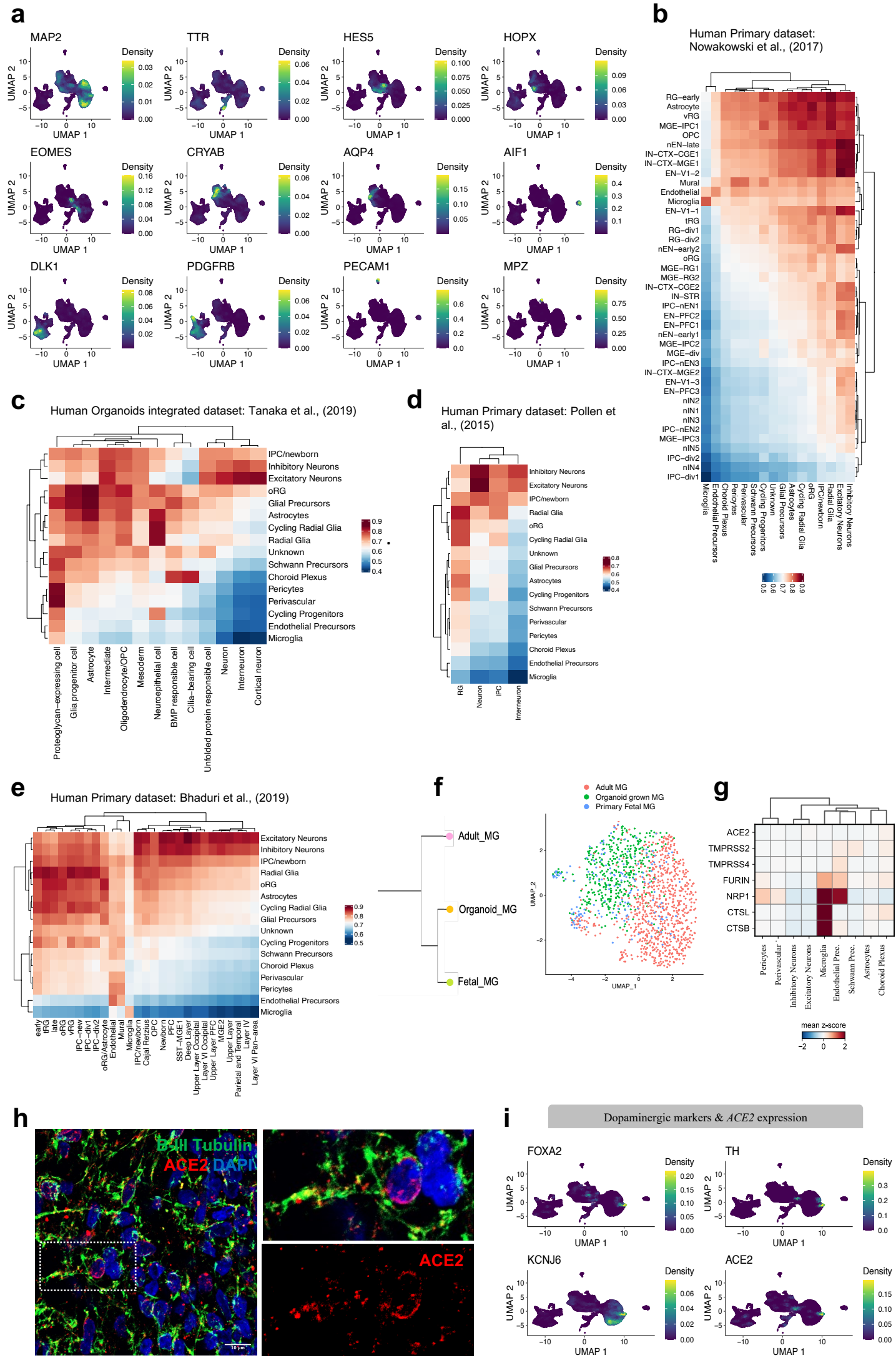

Supplement: Supplementary file 7 — Supplementary Figure 6 [file 41380_2022_1786_MOESM7_ESM.pdf]

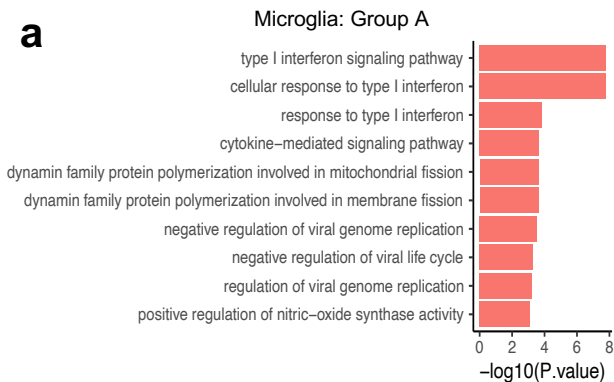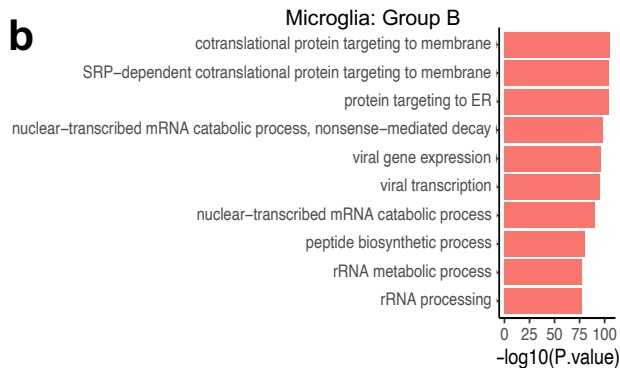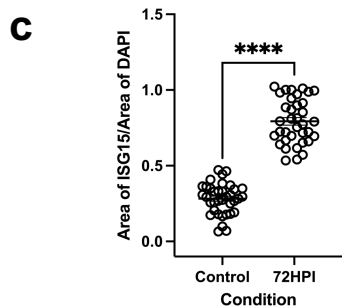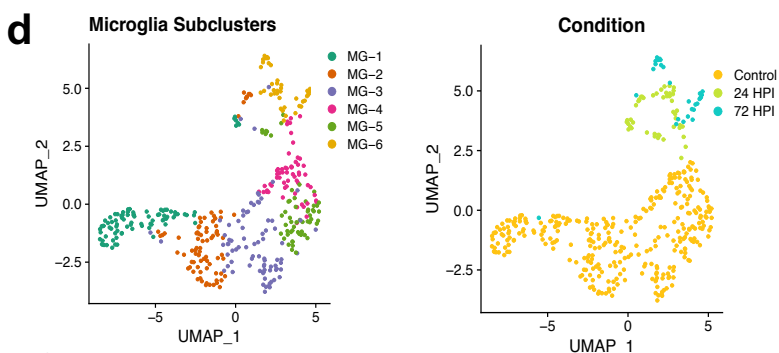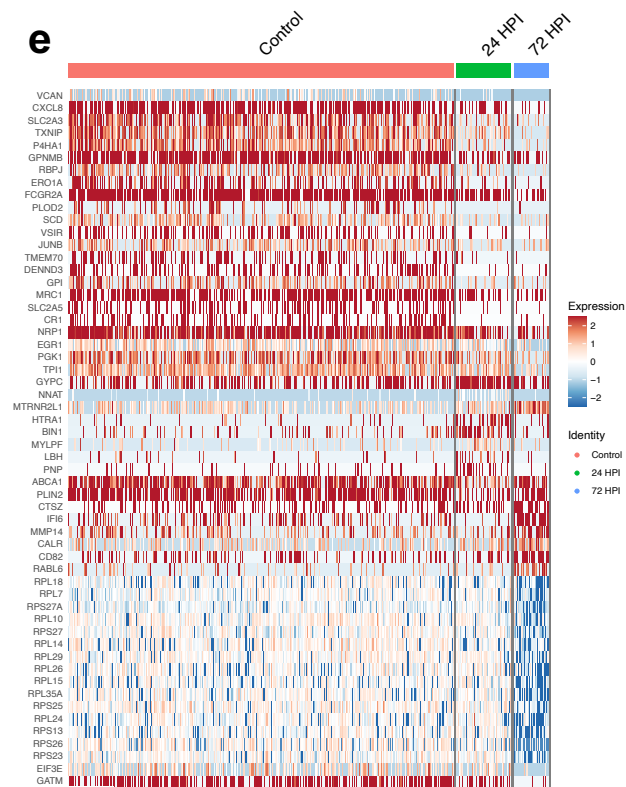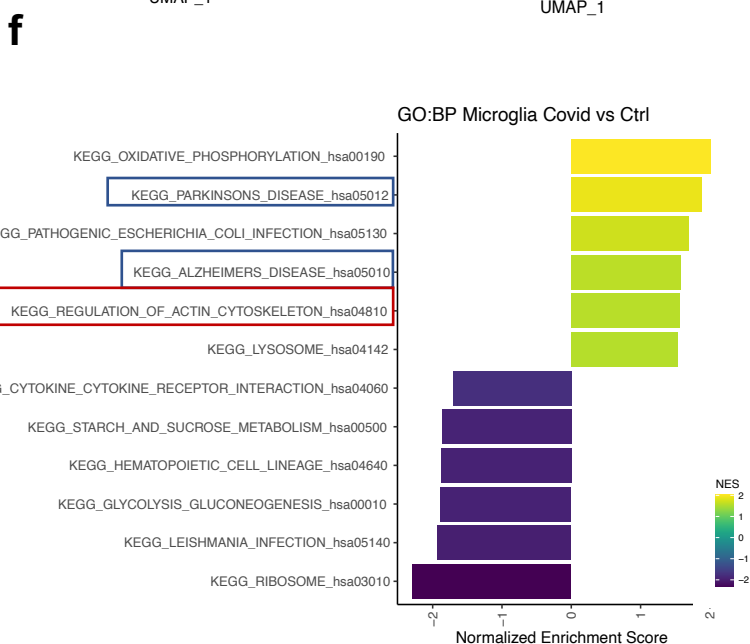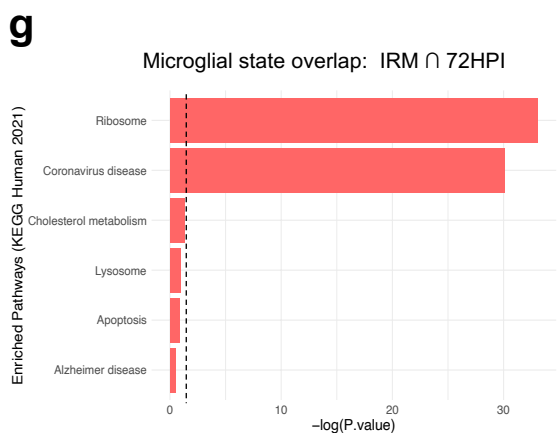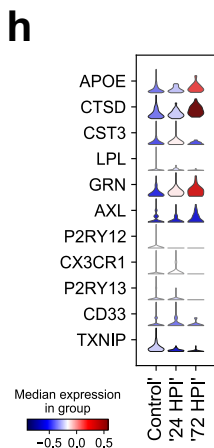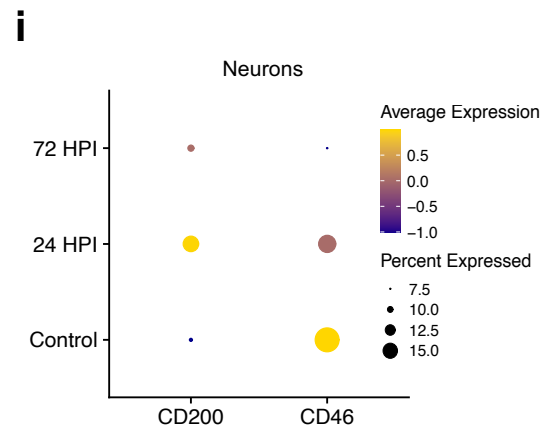

Supplement: Supplementary file 8 — Supplementary Figure 7 [file 41380_2022_1786_MOESM8_ESM.pdf]

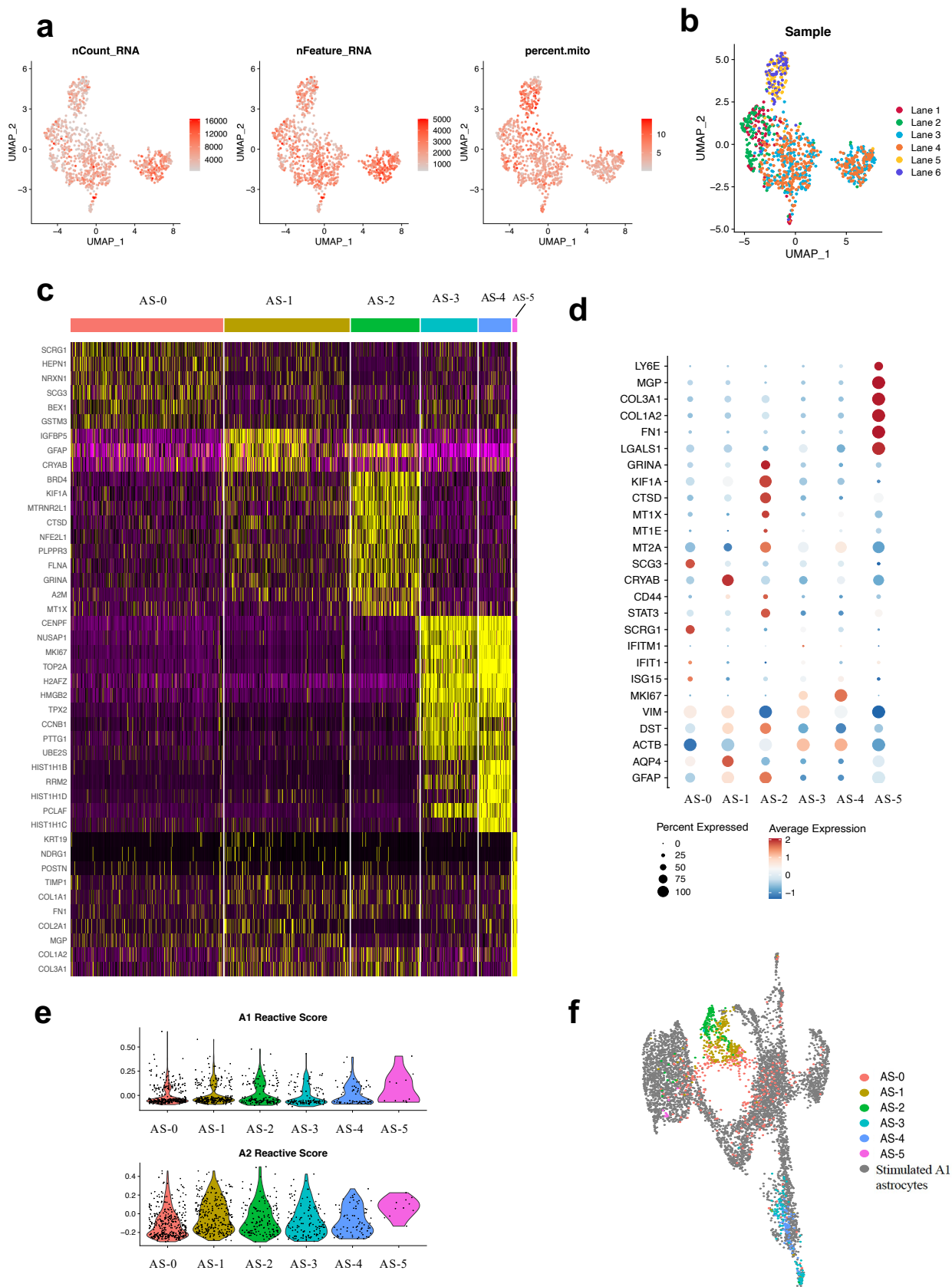

Supplement: Supplementary file 9 — Supplementary Figure 8 [file 41380_2022_1786_MOESM9_ESM.pdf]

**a**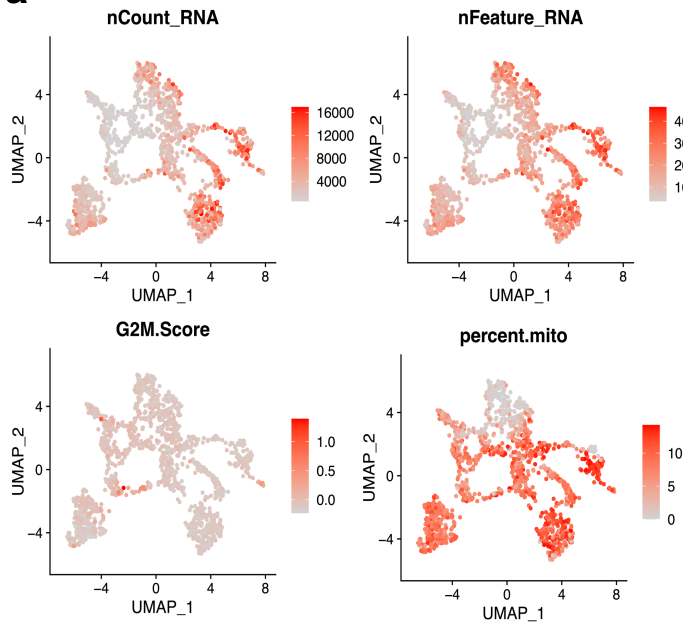**b**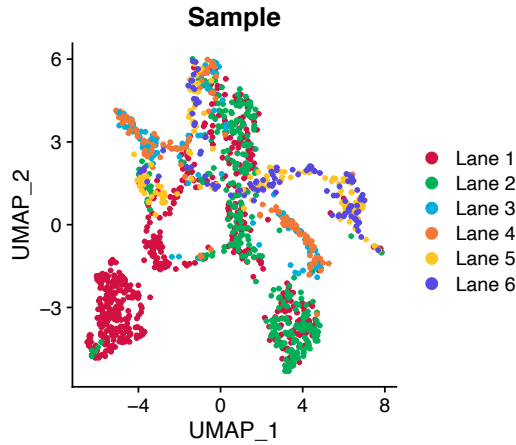**c**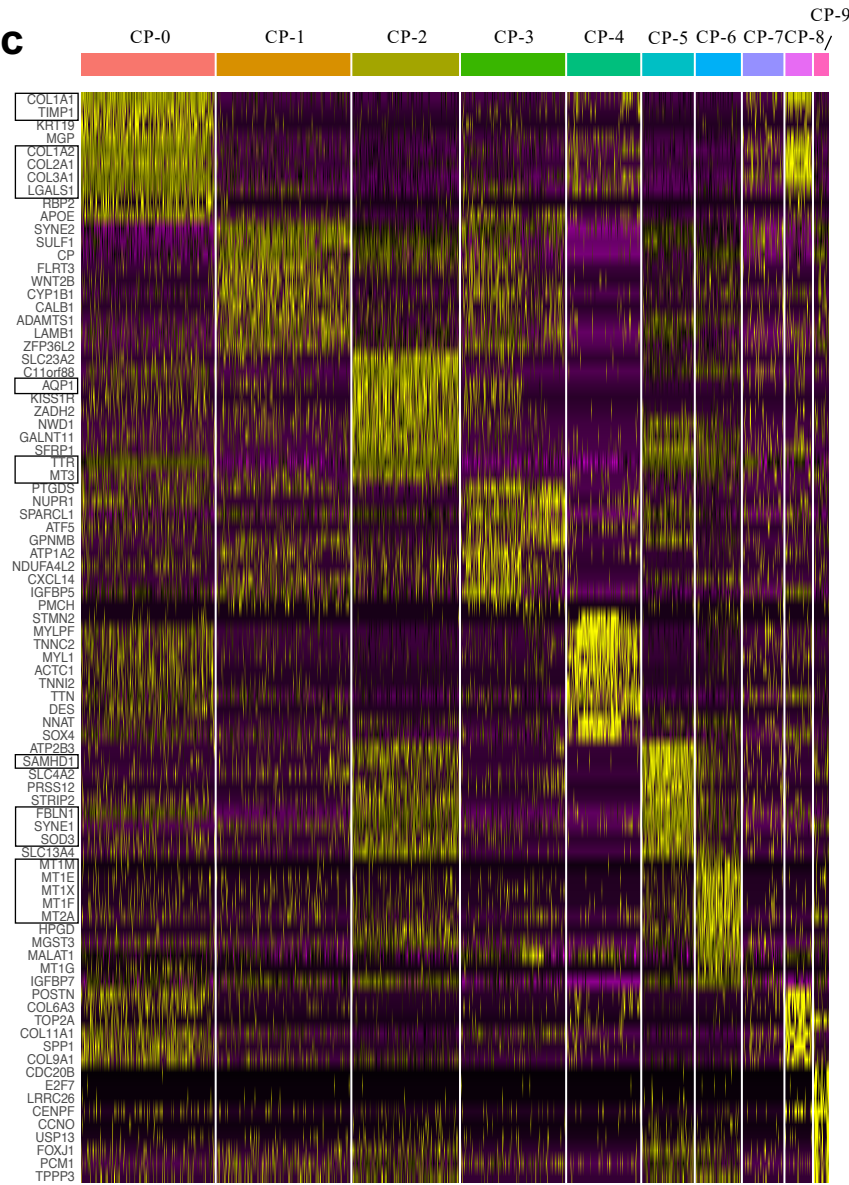

Supplement: Supplementary file 10 — Supplementary Figure 9 [file 41380_2022_1786_MOESM10_ESM.pdf]
