# Supplementary material for: SARS-CoV-2 promotes microglial synapse elimination in human brain organoids
Source: Mol Psychiatry. 2022 Oct 5;27(10):3939–50. doi: 10.1038/s41380-022-01786-2 (PMC9533278; doi:10.1038/s41380-022-01786-2)
Supplement: Supplementary file 6 — Supplementary Figure 5 [file 41380_2022_1786_MOESM6_ESM.pdf]

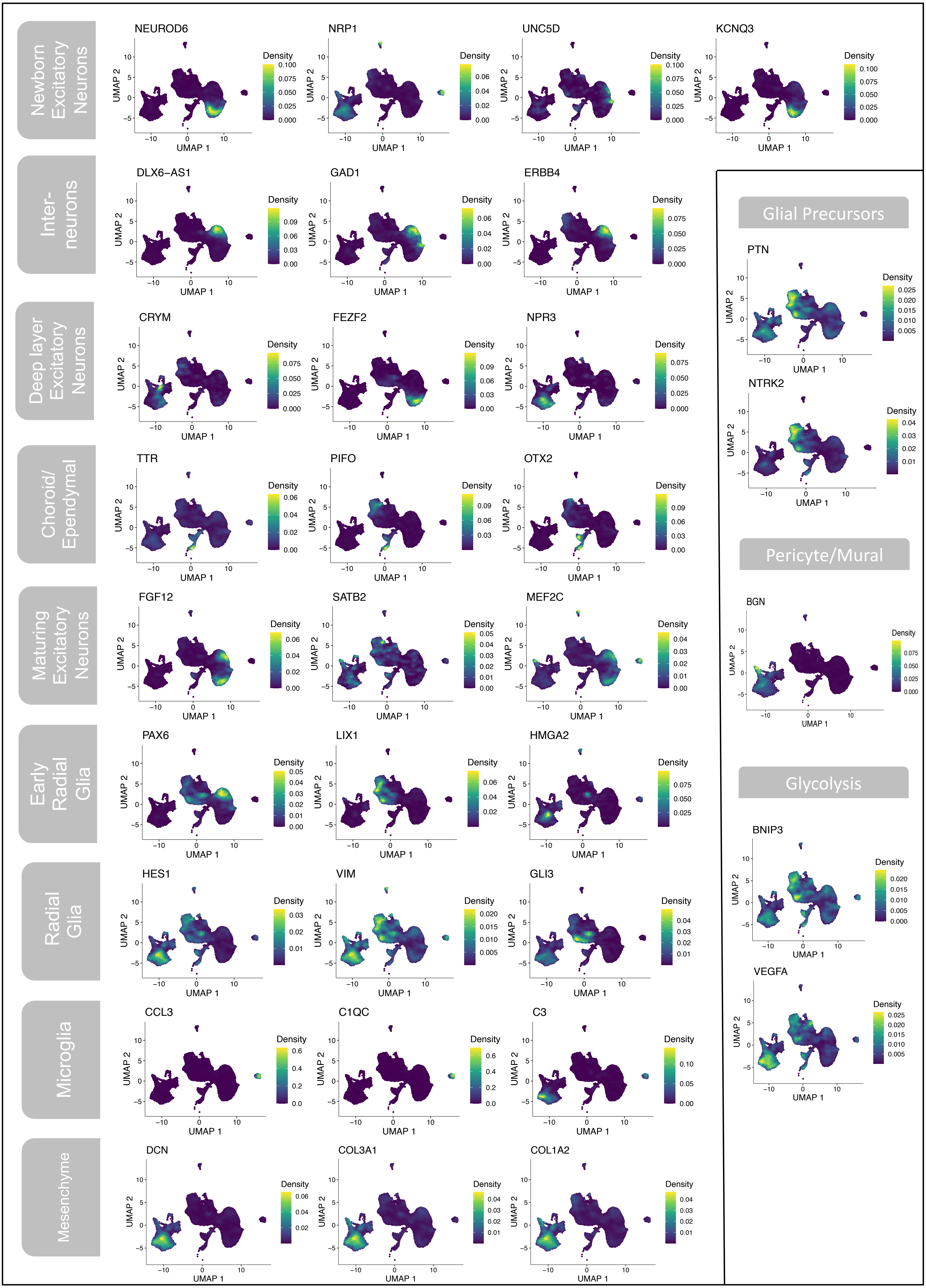

Newborn Excitatory Neurons
NEUROD6
NRP1
UNC5D
KCNQ3
Inter-neurons
DLX6-AS1
GAD1
ERBB4
Deep layer Excitatory Neurons
CRYM
FEZF2
NPR3
Choroid/Ependymal
TTR
PIFO
OTX2
Maturing Excitatory Neurons
FGF12
SATB2
MEF2C
Early Radial Glia
PAX6
LIX1
HMGA2
Radial Glia
HES1
VIM
GLI3
Microglia
CCL3
C1QC
C3
Mesenchyme
DCN
COL3A1
COL1A2
Glial Precursors
PTN
NTRK2
Pericyte/Mural
BGN
Glycolysis
BNIP3
VEGFA
Density
UMAP 1
UMAP 2
